# Resistance to Extreme Stresses by a Newly Discovered Japanese Tardigrade Species, *Macrobiotus kyoukenus* (Eutardigrada, Macrobiotidae)

**DOI:** 10.3390/insects13070634

**Published:** 2022-07-15

**Authors:** Michele Cesari, Ilaria Giovannini, Tiziana Altiero, Roberto Guidetti, Richard Cornette, Takahiro Kikawada, Lorena Rebecchi

**Affiliations:** 1 Department of Life Sciences, University of Modena and Reggio Emilia, Via G. Campi 213/d, 41125 Modena, Italy; michele.cesari@unimore.it (M.C.); roberto.guidetti@unimore.it (R.G.); lorena.rebecchi@unimore.it (L.R.); 2 Department of Education and Humanities, University of Modena and Reggio Emilia, Viale Timavo, 93, 42121 Reggio Emilia, Italy; tiziana.altiero@unimore.it; 3 Division of Biomaterial Sciences, National Institute of Agrobiological Sciences, NARO, 1-2 Owashi, Tsukuba 305-0851, Ibaraki, Japan; cornette@affrc.go.jp (R.C.); kikawada@affrc.go.jp (T.K.)

**Keywords:** integrated taxonomy, phylogeny, ultraviolet resistance, desiccation tolerance, freezing tolerance, sensory field

## Abstract

**Simple Summary:**

Tardigrades are small micrometazoans able to resist several environmental stresses in any stage of their life cycle. The integrated molecular and morphological analysis of tardigrade specimens collected in Tsukuba (Japan) revealed that this population represents a new species, *Macrobiotus kyoukenus* sp. nov., belonging to the genus *Macrobiotus*, one of the most speciose and widespread water bear taxon. The stress resistance capabilities of *M. kyoukenus* sp. nov. have been tested by submitting animals to extreme desiccation, rapid freezing, and high levels of ultraviolet radiations (UVB and UVC). Animals were able to survive desiccation and freezing, and both hydrated and desiccated animals showed a high tolerance to increasing UV radiations. Overall, our findings contribute to the discovery of a larger tardigrade biodiversity in Japan, and the tolerance capabilities of *M. kyoukenus* sp. nov. show that this new species could become an emerging model for stress resistance studies.

**Abstract:**

Tardigrades are small micrometazoans able to resist several environmental stresses in any stage of their life cycle. An integrated analysis of tardigrade specimens collected in Tsukuba (Japan) revealed a peculiar morphology and a new sensory field in the cloaca. Molecular taxonomy and phylogenetic analysis on different genes (COI, ITS2, 18S and 28S) confirmed that this population is a new species, *Macrobiotus kyoukenus* sp. nov., belonging to the widespread *Macrobiotus hufelandi* group. The stress resistance capabilities of *M. kyoukenus* sp. nov. have been tested by submitting animals to extreme desiccation, rapid freezing, and high levels of ultraviolet radiations (UVB and UVC). Animals were able to survive desiccation (survivorship 95.71 ± 7.07%) and freezing up to −80 °C (82.33 ± 17.11%). Both hydrated and desiccated animals showed a high tolerance to increasing UV radiations: hydrated animals survived to doses up to 152.22 kJ m^−2^ (UVB) and up to 15.00 kJ m^−2^ (UVC), while desiccated specimens persisted to radiations up to 165.12 kJ m^−2^ (UVB) and up to 35.00 kJ m^−2^ (UVC). Present data contribute to the discovery of a larger tardigrade biodiversity in Japan, and the tolerance capabilities of *M. kyoukenus* sp. nov. show that it could become a new emerging model for stress resistance studies.

## 1. Introduction

Water bears are small hygrophilous metazoans belonging to the phylum Tardigrada inhabiting marine, freshwater and terrestrial environments. They are able to survive when surrounded by a film of water, and therefore they can colonize terrestrial environments such as mosses, lichens, soil and leaf litter, in most cases as interstitial organisms. This capability is linked to their ability to enter cryptobiosis at any stage of their life cycle, from egg to adult [1]. Cryptobiosis includes several strategies such as anhydrobiosis, cryobiosis, anoxybiosis, and osmobiosis, respectively, induced by desiccation, cooling, lack of oxygen and extreme high level of solutes in the surrounding environment [2]. To perform cryptobiosis, tardigrades stop their metabolism and enter a state of suspended life, which can be reverted when favourable conditions return (for a review see [1,3]). Moreover, tardigrades can withstand several physical and chemical extremes (e.g., very low and high temperature, high pressure, vacuum, organic solvents and radiations) not only when they are in desiccated state, but also when active [2,3,4,5,6,7,8]. In particular, tardigrades are able to withstand extreme levels of gamma, X-ray, ionizing and ultraviolet radiations, with the tested experimental conditions far more extreme than those imposed by their natural habitats, including outer space [4,9,10,11,12,13].

More than 1400 species of tardigrades are known, with the parachelan *Macrobiotus* C.A.S. Schultze, 1834 (Eutardigrada, Macrobiotidae) being one of the most speciose genera and with a worldwide distribution [14,15]. However, this genus is represented in Japan by five species only: *Macrobiotus echinogenitus* Richters, 1904 [16,17,18], *Macrobiotus hufelandi* C.A.S. Schultze, 1834 [16,19,20,21], *Macrobiotus occidentalis* Murray, 1910 [16,17,19,20,22,23,24], *Macrobiotus shonaicus* Stec et al. 2018 [25,26], and *Macrobiotus topali* Iharos, 1969 [20,24]. Inside the genus *Macrobiotus*, the *Macrobiotus hufelandi* species group (i.e., species with animals with two macroplacoids and a microplacoid in the muscle pharynx, three bands of teeth in the oral cavity, and pores on their cuticle; [27,28]) is the most diverse and widespread. Conversely, this species group is represented in Japan only by the nominal species, *Macrobiotus hufelandi*, and the recently described *Macrobiotus shonaicus*. Considering that most findings are starting to be outdated and that the status of some species are now disputed (see for example *M. topali* by [29]), the present situation highlights that unfortunately Japanese tardigrade biodiversity is still understudied.

The sampling of a moss in Tsukuba (Japan) allowed us to retrieve and identify a new tardigrade species. The integrated morphological (light microscopy, scanning electron microscopy, and morphometric data) and molecular analyses (18S rRNA, 28S rRNA, ITS-2 nuclear DNA, and *cox1* mitochondrial DNA) confirmed its species status and pointed out its belonging to the *Macrobiotus hufelandi* species group. Furthermore, its stress resistance capability to different environmental stresses was tested by submitting animals to extreme desiccation, rapid freezing, and high levels of ultraviolet radiation.

## 2. Materials and Methods

A moss sample was collected on the trunk of a Japanese *Zelkova serrata* (Thunb.) Makino (1903) (zelkova tree) in the garden of the National Institute of Agrobiological Sciences (NIAS) in Tsukuba, Ibaraki prefecture (Japan; geographical coordinates: 36° 3.168′ N; 140° 5.419′ E; 25 m a.s.l.) on 26 April 2018. The sample was soaked in tap water for half an hour, and afterwards tardigrades and their eggs were extracted from the sample by washing and squeezing the moss fragment through consecutive 500 μm and 38 μm sieves. Tardigrades and eggs were individually isolated using a needle and a glass pipette under a stereomicroscope (Leica MZ125). Remaining fractions of the sample are stored at −80 °C in the Biobank of the Evolutionary Zoology Lab at the Department of Life Sciences (University of Modena and Reggio Emilia, Italy—UNIMORE) for future studies.

Tardigrades (45 specimens) and eggs (11) were mounted in Faure-Berlese fluid for observations under light microscopy (LM), using Phase Contrast (PhC) or Differential Interference Contrast (DIC), while other animals (10) and eggs (10) were prepared for scanning electron microscopy (SEM), following the protocol described in Bertolani et al. [30]. Some isolated eggs were maintained in water in a small container until hatching, then newborns were processed for molecular analyses (see below), while the egg shell was mounted in Faure-Berlese fluid to establish the relationship between morphology and sequences. Additional specimens (51 adults) were stained with acetic lactic orcein for determination of gender, presence of a seminal receptacle and karyotype. Two adults and seven newborns were used for molecular analysis (for more details see below). The LM observations were carried out up to maximum magnification (objective 100× with oil immersion) with a Leica DM RB microscope, equipped with an AmScope MU1803 digital camera. Morphometric data of animal and egg structures were according to Kaczmarek & Michalczyk [31], and handled using the ‘Parachela’ ver. 1.6 template (available from the Tardigrada Register [32]), updated with the Thorpe’s normalization of the data (as in Massa et al. [33] according to Bartels et al. [34]). Morphometric data are available in Table S1. The SEM observations were carried out with a Nova Nano SEM 450 (FEI Company—Oxford Instruments, Hillsboro, OR; USA), available at the ‘Centro Interdipartimentale Grandi Strumenti’ at UNIMORE. Slides are deposited in Bertolani’s collection (Department of Life Sciences, UNIMORE).

Total genomic DNA was extracted from single adult tardigrades and from newborns. The extractions were performed with QuickExtract™ DNA Extraction Solution (Lucigen, Middleton, WI, USA), following the manufacturer’s protocol. All animals were previously observed in vivo up to 100× oil immersion magnification to avoid mistakes in determining the morphology. Each specimen was identified and photographed in vivo with LM, using the method described by Cesari et al. (2011) in order to obtain voucher specimens. Four DNA fragments were amplified: the small ribosome subunit (18S rRNA), the large ribosome subunit (28S rRNA), the internal transcribed spacer (ITS-2), and the cytochrome oxidase subunit I (*cox1*), using the primers and protocols described by Bertolani et al. [35], Cesari et al. [36], Stec et al. [37] and Bertolani et al. [38], respectively. The amplified products were gel purified using the Wizard Gel and PCR cleaning (Promega) kit, while sequencing reactions were performed using the ABIPRISM^®^ BigDye™ Terminator Version 1.1 Sequencing Kit (Applied Biosystems, Foster City, CA, USA) on purified amplicons. Each sequencing reaction contained 0.2 μM of a single PCR primer to initiate the sequencing reaction, 2 μL of BigDye™, 70 ng of purified products, 4 μL of 5× BigDye™ Terminator Version 1.1 Sequencing Buffer (Applied Biosystems, Foster City, CA, USA) and bi-distilled H_2_O for a final volume of 20 μL. Cycling conditions for sequencing reactions consisted of 25 cycles of 96 °C for 10 s, 50 °C for 5 s and 60 °C for 4 min. Both strands were sequenced using an ABI Prism 3100 (Applied Biosystems, Foster City, CA, USA). Nucleotide sequences of the newly analysed specimens were submitted to GenBank (accession numbers: ON809460-5 for *cox1* gene; ON818300-5 for the ITS-2 gene; ON818306-11 for the 28S gene and ON818312-6 for the 18S gene, Table 1).

The phylogenetic analysis was carried out on 18S and 28S genes. Nucleotide sequences were aligned with the MAFFT algorithm [39], as implemented in the MAFFT online service [40], and checked by visual inspection. Sequences pertaining to *Ramazzottius varieornatus* Bertolani and Kinchin, 1993 total genome (Eutardigrada, Hypsibioidea, GenBank acc. no.: BDGG01000030) were used as an outgroup. Other Macrobiotoidea sequences from GenBank were also included in the analysis (Table S2). A phylogenetic analysis of the dataset was computed in a maximum likelihood (ML) framework, using the program RAxML version 8.2.12 [41] as implemented in CIPRES. The evolutionary model was inferred on 18S and 28S genes using the Corrected Akaike Information Criterion implemented in jModeltest 2.1.10 [42,43] and resulted to be GTR+I+G. However, due to the strong correlation between invariant sites ‘I’ and gamma distribution ‘G’ [44], the GTR+G model was utilized. Bootstrap resampling with 1000 replicates was undertaken via the rapid bootstrap procedure of Stamatakis et al. [45] to assign support to branches in the ML tree. 

For species delimitation analysis, *cox1* and ITS-2 sequences were considered. Nucleotide sequence divergences between scored haplotype genes were computed using p-distance by utilizing MEGAX [46]. The relationships among *cox1* sequences were estimated using a haplotype parsimony network by applying the method described by Templeton et al. [47], as implemented in TCS 1.21 [48] and visualized using tcsBU [49]. A 95% connection limit was employed because it has been suggested as a useful general tool in species assignments and discovery [50]. Species delimitation was also inferred by using the Assemble Species by Automatic Partitioning method (ASAP; [51]) and the Poisson Tree Process (PTP; [52]). The distance-based ASAP analysis was performed on the ASAP website (https://bioinfo.mnhn.fr/abi/public/asap/, accessed on 6 June 2022). The PTP was inferred by using a starting maximum likelihood (ML) gene tree computed using RAxML as implemented in CIPRES, under the GTR+G model, as inferred by using the Akaike Information Criterion on jModelTest2. A sequence of *Paramacrobiotus richtersi* (Murray, 1911) (Eutardigrada, Macrobiotoidea; GenBank acc. no.: MK040992) was used as an outgroup. Bootstrap resampling (1000 replicates) was undertaken as described above.

### Stress Resistance

To test the ability of *M. kyoukenus* sp. nov. to withstand stress conditions such as desiccation, freezing, UVB and UVC radiation, animals in three different physiological states, namely hydrated (active), desiccated (anhydrobiotic), and frozen (cryobiotic) were considered. To standardize the experimental conditions, before starting the experiments the specimens were starved for 24 h in natural mineral water (pH 7.5; 34.10 mg L^−1^ Ca^2+^) at 15 °C.

To analyze the tolerance capability of *M. kyoukenus* sp. nov. to desiccation, six replicates of 10 animals each were used. Animals were desiccated under laboratory conditions, using the slightly modified protocol of Rebecchi et al. [53]. Each group of animals was placed on Whatman filter paper with a few drops of natural mineral water and maintained in a climatic chamber at the following temperature and relative humidity (RH): 18 °C at 80% RH for 4 h, 18 °C at 50% RH for 4 h, and room temperature at 0 to 3% RH for 2 days. Successively, the animals were rehydrated by adding water drops to each filter paper every 10 min for a total of 60 min. After rehydration, coordinated movements of the animal body (locomotion performance) constituted the criterion to confirm animal viability [53]. Locomotion performance was evaluated immediately after rehydration (t_0_), 1 h (t_1_) and 24 h (t_24_) later. During rehydration, animals were kept in plastic boxes containing natural mineral water at 15 °C with a 12 h/12 h (light/dark) photoperiod. The term ‘final survival’ refers to the survival rate of animals recorded at t_24_. The Kruskal-Wallis test was applied to compare the percentages of viable tardigrades (with locomotion performance) at t_0_, t_1_ and t_24_. All statistical analyses were carried out using SPSS 28 (SPSS Inc., Chicago, IL, USA).

To test the capability of *M. kyoukenus* sp. nov. to tolerate freezing, six replicates of 10 animals each were used. Groups of hydrated tardigrades were cooled in 4 mL of natural mineral water within plastic vials (2 × 3 cm; diameter × height). Plastic vials containing tardigrades in water were frozen starting from 14 °C down to a constant temperature of −9 °C, −20 °C or −80 °C [54]. Cooling rates to reach the three different temperatures were −0.24 °C min^−1^ at −9 °C, −0.5 °C min^−1^ at −20 °C, and −2.3 °C min^−1^ at −80 °C, recorded by a thermocouple (Vernier Software & Technology, Beaverton, OR, USA). Tardigrades were held frozen at the target temperature for six days. Then, plastic vials with animals held at −20 °C and −80 °C were transferred to −9 °C for 15 h to ensure the same thawing process for all experimental conditions. Thereafter, tardigrades were thawed at 15 °C and the locomotion performances were evaluated at 2.5 h (t_2.5_) post thawing and after 24 h (t_24_, final survival; [54]). The Kruskal-Wallis test was applied to compare the percentages of viable animals at t_2.5_ and t_24_ after freezing at the three temperatures.

Specimens were exposed to UVB through a wide spectrum of UV radiation with a peak of emission at 312 nm (UVB). The UV source consisted of a transilluminator 15 W bulb (Sigma-Aldrich, St Louis, MO, USA) 40 cm in length. Midrange UV fluorescence λ^em^ 312 nm yielded a spectral output extending from the UVC through the UVA spectra with an emission peak at 312 nm (see [9,12]). The irradiance, as measured with a spectroradiometer (Macam SR9910, Macam Photometrics, Livingstone, UK), was 0.26 kJ m^−2^ min^−1^. The lamp was positioned 30 cm above the samples, both placed in a climatic chamber in dark conditions at 15 °C (see [9,12]). Animals were exposed to sixteen UVB doses: 2.58 kJ m^−2^, 5.16 kJ m^−2^, 10.32 kJ m^−2^, 23.22 kJ m^−2^, 36.12 kJ m^−2^, 49.02 kJ m^−2^, 61.92 kJ m^−2^, 74.82 kJ m^−2^, 87.72 kJ m^−2^, 100.62 kJ m^−2^, 113.42 kJ m^−2^, 126.42 kJ m^−2^, 139.32 kJ m^−2^, 152.22 kJ m^−2^, 165.12 kJ m^−2^, 178.02 kJ m^−2^.

UVC radiation stress experiments were performed using the HL-2000 HybriLinkerTM Hybridization Oven combining an HB-1000 Hybridization oven and UV Crosslinker (254 nm UV). Animals were exposed to twelve UVC doses: 2.5 kJ m^−2^, 5.0 kJ m^−2^, 6.0 kJ m^−2^, 7.0 kJ m^−2^, 8.0 kJ m^−2^, 9.0 kJ m^−2^, 10.00 kJ m^−2^, 15.00 kJ m^−2^, 23.22 kJ m^−2^, 30.00 kJ m^−2^, 35.00 kJ m^−2^, 40.00 kJ m^−2^.

The UVB and UVC radiation tolerances of *M. kyoukenus* sp. nov. were evaluated on active and desiccated animals. Two replicates of 20 animals were used for each physiological condition and UV dose. Hydrated animals were exposed to UV radiation within a thin layer of natural mineral water (350 μL) in a small plastic Petri dish (1 × 0.7 cm; diameter × height) without the cover, whereas desiccated tardigrades were irradiated on the same Whatman filter paper on which they were dried [9,12]. The desiccation protocol was described above. During the irradiation, the temperature was kept at 15 °C. For non-irradiated controls, two replicates each of 20 hydrated or desiccated animals were kept in a covered and shielded box within the climatic chamber or hybridization oven containing the UVB and UVC sources, respectively. The criterion of locomotion performance was used to evaluate tardigrade viability after irradiation [9,12]. The locomotion performance of hydrated animals was recorded immediately after the end of irradiation (t_0_), and 1 h (t_1_) and 24 h (t_24_; final survival) later. Desiccated tardigrades were rehydrated immediately after irradiation (see above), and their locomotion performance was recorded at the end of rehydration (t_0_), after 1 h (t_1_) and 24 h (t_24_) from the end of rehydration. During these procedure phases, tardigrades were kept in the dark at 15 °C. The Kruskal-Wallis test was used to compare the percentages of viable animals among t_0_, t_1_ and t_24_ after the exposition of both active and desiccated animals to UVB or UVC. The Spearman correlation test was used to verify the hypothesis that the final survival of the hydrated or desiccated animals declines with the increase in the UVB or UVC radiation doses. To calculate the lethal doses that cause 50% mortality (LD_50_) of the hydrated and desiccated specimens at t_24_ after the exposition to UVB or UVC, the Probit analysis was used. Moreover, the comparison among the final survivals (t_24_) of both active and desiccated animals irradiated with UVB and UVC was carried out using the Kruskal-Wallis test.

## 3. Results

### 3.1. Morphological Analyses

*Macrobiotus kyoukenus* sp. nov.

ZOOBANK: urn:lsid:zoobank.org:act:E1DEC85D-BB12-43F0-8D57-7F8C252B3DA2

*Type locality*: 36° 3.170′ N, 140° 5.420′ E; 25 m a.s.l., Japan, Ibaraki Prefecture, Tsukuba, garden of National Institute of Agrobiological Sciences; moss on *Zelkova serrata* (Thunb.) Makino tree; collected in April 2018.

*Type repositories*: The holotype (C4313-2), 105 paratypes (44 mounted in Faure-Berlese fluid, 51 in orcein, 10 prepared for SEM analyses), and 21 eggs (11 mounted in Faure-Berlese fluid, 10 for SEM analyses) are in the Bertolani Collection of UNIMORE.

*Etymology*: The name of the species derives from the Japanese word 強健 (kyouken), meaning ‘robust; strong; sturdy’, referring to its high stress resistance capabilities.

*Description* (morphometric data in Table S1)

**Figure 1:**
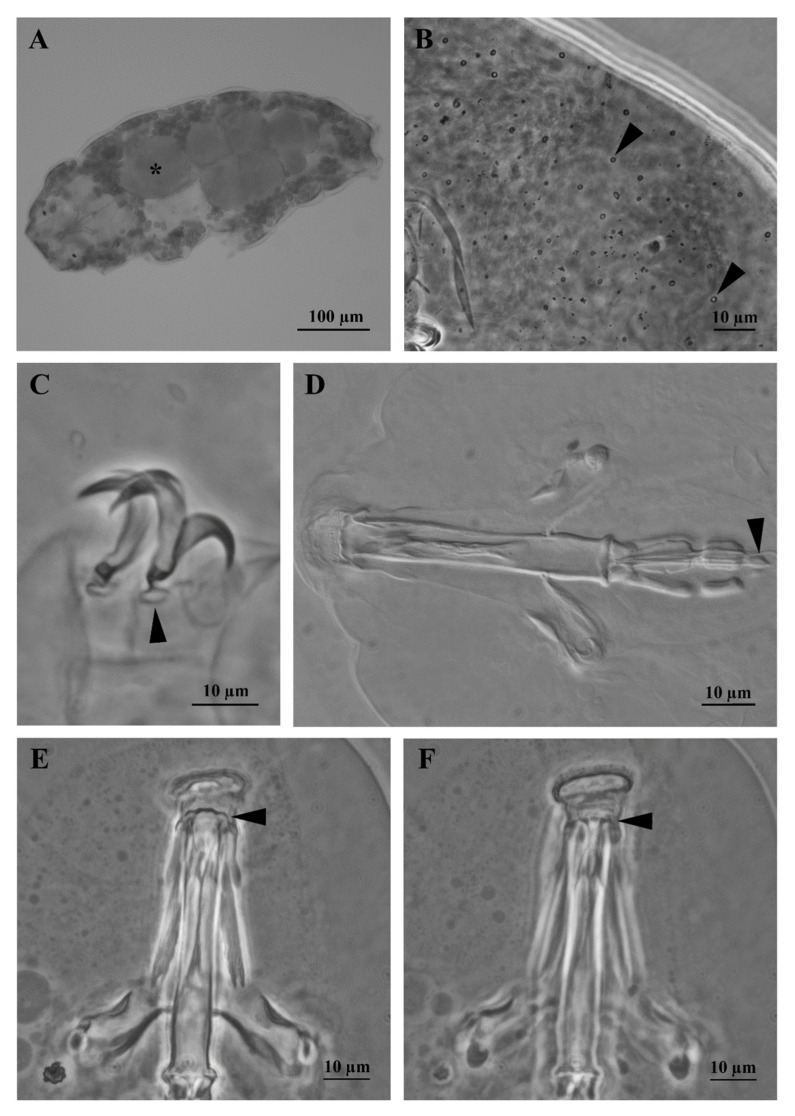
*Macrobiotus kyoukenus* sp. nov. (**A**) *In toto* and in vivo female specimen, asterisk denotes an egg; (**B**) Cuticular pores (arrowheads) after orcein staining; (**C**) Claws of the second pair of legs with lunules (arrowhead); (**D**) Buccal pharyngeal apparatus with two macroplacoids and one microplacoid (arrowhead); (**E**) Dorsal transversal crests of the buccal armature (arrowhead); (**F**) Ventral transversal crests of the buccal armature (arrowhead). (**A**–**C**,**E**,**F**) PhC; (**D**) DIC.

**Figure 2:**
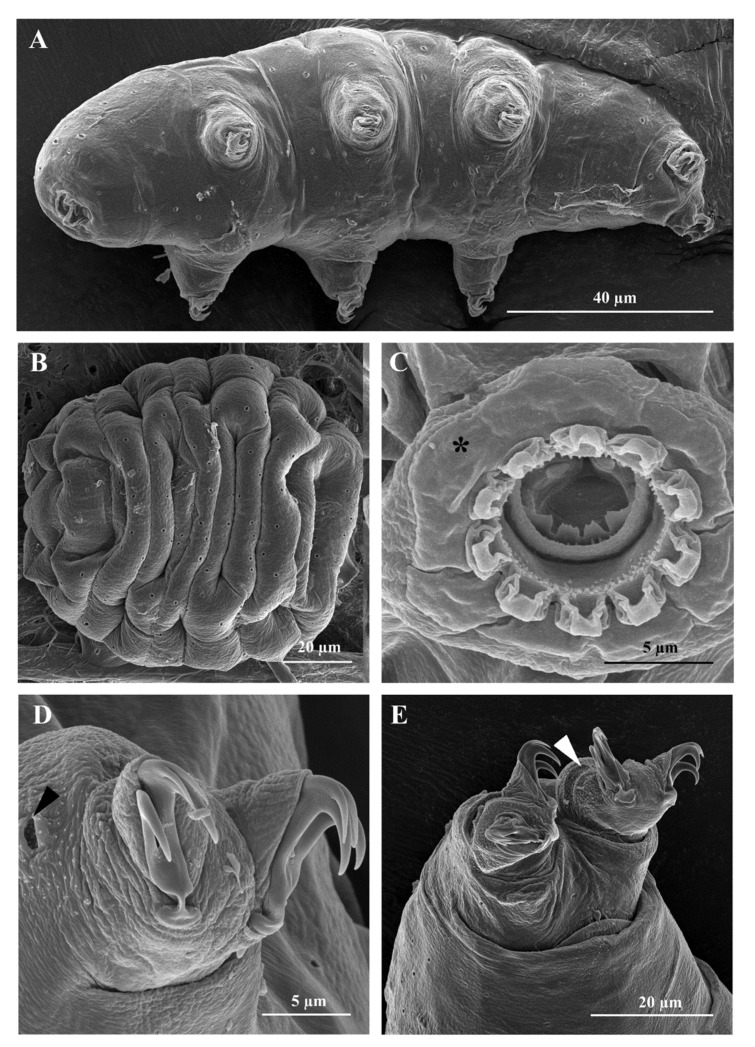
*Macrobiotus kyoukenus* sp. nov. (**A**) *In toto* specimen; (**B**) Desiccated *in toto* specimen; (**C**) Mouth opening with circum-oral sensory field (COS) (asterisk); (**D**) Claws of the second pair of legs with a pore showing an internal granulation (arrowhead); (**E**) Claws of the fourth pair of legs ornamented with a patch of fine cuticular granules (arrowhead). (**A**–**E**) SEM.

**Figure 3:**
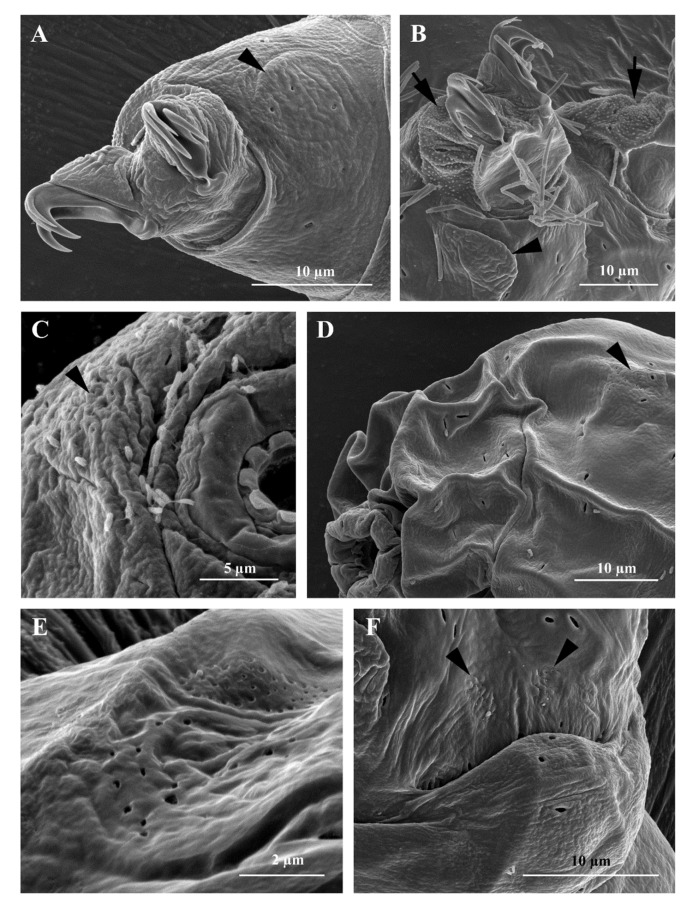
*Macrobiotus kyoukenus* sp. nov. (**A**) Third pair of legs with claws and a flat bulge (arrowhead); (**B**) Fourth pair of legs ornamented with cuticular granules (arrows) and with claws and a flat bulge (arrowhead); (**C**) Antero-lateral sensory field (ALS) with small pores on the side of the mouth (arrowhead); (**D**) Postero-lateral sensory field (PLS) with small pores on the side of the head (arrowhead); (**E**) Postero-lateral sensory field with small pores on the side of the head (enlargement of d); (**F**) Cloaca sensory fields with small pores (arrowheads). (**A**–**F**) SEM.

Body: white-yellowish, from 107.0 to 395.9 µm in length (Figure 1A). Eye-spots present (Figure 1A). Smooth cuticle, with oval pores of different size (0.4 to 2.0 µm in longer diameter; Figure 1B and Figure 2A), randomly distributed on all body surface (Figure 1B). Larger pores with an internal granulation (Figure 2D). First three pairs of legs ornamented on the external side with a patch of fine cuticular granules (not always visible with LM), composed by small cones (visible only with SEM; Figure 2D), within this patch a large pore is present (Figure 2D). Posterior and lateral sides of hind legs covered with larger granules (Figure 2E). A flat bulge present on the internal side of the three anterior legs and on the external side of each hind leg (with very small pores on its surface, visible only with SEM; Figure 3A,B). With SEM, areas with small pores present on each side of the mouth (Figure 3C), of the head (Figure 3D,E), and close to the cloaca (Figure 3F), corresponding to the “antero-lateral”, “postero-lateral”, and “cloaca” sensory fields, respectively (see Discussion).

Bucco-pharyngeal apparatus (Figure 1D) with antero-ventral mouth surrounded by a ring of smooth cuticle apparently not organized in lobes, corresponding to the “circum-oral” sensory field and 10 peribuccal lamellae (Figure 2C). Buccal armature: anterior (first) and posterior (second) bands of teeth visible with LM only in larger specimens (i.e., longer than 380 µm; Figure 1D and Figure 3C); the thin dorsal transversal crests in contact between them (sometime appearing as a continuous line with LM, but still distinguishable with SEM; Figure 1E and Figure 3D); the thin ventral transversal crests well separated and large (Figure 1F). Wide buccal tube with ventral lamina. Stylet supports in shape of an elongated sigma with a distal flat enlargement, inserted at 70.4 to 79.4% of the buccal tube. Typically-shaped stylet furcae, with large drop-shaped condyles supported by short branches provided with rounded apophyses. In the pharynx: large and triangular pharyngeal apophyses, two rod-shaped (in lateral view) macroplacoids (the first the longest), and a small and thin microplacoid (Figure 1D). In frontal view, first macroplacoid with a light median constriction (deeper in larger specimens), second macroplacoid rectangular (with a light subterminal constriction only in larger specimens) (Figure 1D). Double-claws of *hufelandi* type (Figure 1C, Figure 2D,E and Figure 3A,B), primary branches with large accessory points. Small and smooth lunules in claws I-III, larger in claws IV (Figure 1C and Figure 2E).

#### 3.1.1. Reproduction

**Figure 4:**
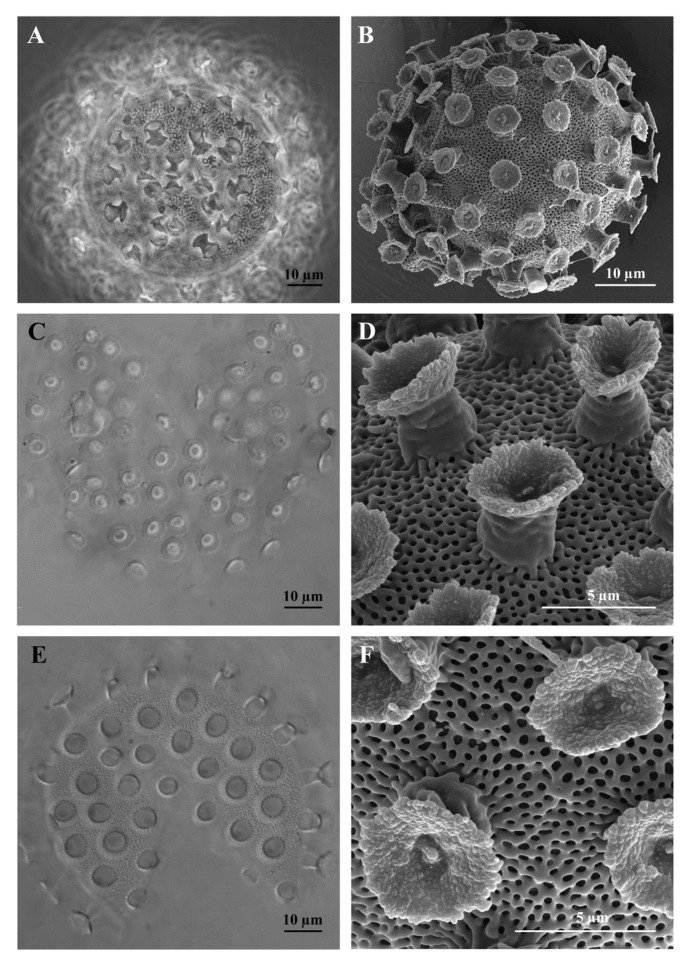
Eggs of *Macrobiotus kyoukenus* sp. nov. (**A**) *In toto* egg; (**B**) *In toto* egg; (**C**) Terminal disks of the egg processes; (**D**) Terminal disks of the egg processes; (**E**) Egg surface between the processes; (**F**) Egg surface between the processes. (**A**) PhC; (**B**,**D**,**F**) SEM; (**C**,**E**) DIC.

The species is amphimictic and gonochoric. Sex ratio analysis showed 12 males and 9 females (sex ratio 1.3:1), while it was not possible to identify the sex condition for other 30 individuals. Males have sperms, while no seminal receptacle was observed in females, who produce spherical ornamented eggs (Figure 4A,B), laid freely, 56.3 to 80.5 µm in diameter excluding processes (mean 67.4 µm, SD 6.7 µm; *n* = 9). Processes in the shape of upside down goblets (Figure 4D,E). Following the definitions of Kaczmarek & Michalczyk [31], the processes have a “straight” trunk shape, specifically, conical or almost cylindrical processes (with annulations of their surface visible only with SEM) with a wide terminal disk, “serrated” and “concave” (specifically, with irregular margin and granulated surface with some large or very large granules, mainly in central position; Figure 4B,C), the surface between the processes is of “*hufelandi* type” (specifically, with a net of small meshes of similar size) (Figure 4A,B,D–F). Process height: mean of 5.9 µm (SD 1.2 µm, range 3.7 to 7.9 µm, *n* = 30); process base diameter: mean 6.1 µm (SD 0.9, range 4.0 to 7.4 µm, *n* = 30; % base diameter/height: mean 105% (SD 19%, range 74 to 150%, *n* = 30); distal disc diameter: 5.4 µm (SD 0.9 µm, range 3.9 to 7.6 µm, *n* = 30); % base diameter/disc diameter: mean 115% (SD 21%, 81.8 to 159.5%, *n* = 30). Number of processes on the circumference: mean 18.9 (SD 1.9, range 16 to 22, *n* = 10); number of processes on an egg surface of 1000 µm^2^: 13.3 (SD 2.9, range 10.0 to 18.0, *n* = 10). An egg with a developing embryo was found.

#### 3.1.2. Differential Diagnosis 

Following the taxonomic key of Kaczmarek & Michalczyk [31] and the subsequent bibliography, *Macrobiotus kyoukenus* sp. nov. is similar to the following species: *Macrobiotus canaricus* Stec, Krzywański & Michalczyk, 2018, *Macrobitus dulciporus* Roszkowska, Gawlak, Draga & Kaczmarek, 2019, *Macrobiotus engbergi* Stec, Tumanov & Kristensen, 2020, *Macrobiotus hannae* Nowak & Stec, 2018, *Macrobiotus humilis* Binda & Pilato, 2001, *Macrobiotus kamilae* Coughlan & Stec, 2019, *Macrobiotus nebrodensis* Pilato, Sabella, D’Urso & Lisi, 2017, *Macrobiotus noongaris* Coughlan & Stec, 2019, *Macrobiotus papei* Stec, Kristensen & Michalczyk, 2018, *Macrobiotus sandrae* Bertolani & Rebecchi, 1993, *Macrobiotus sottilei* Pilato, Kiosya, Lisi & Sabella, 2012. *Macrobiotus kyoukenus* sp. nov. differs from the above species in the absence of large lenticular pores (up to 5 µm) on cuticle surface of animals, and in the presence of dorsal transversal crests of the buccal armature in contact with each other (with LM observation), smooth lunules in the hind legs, small meshes (pores) present on the egg surface between processes, egg processes with a larger diameter of the distal disk respect to the basal diameter, a not serrated margin of the distal disk of the egg processes, surface of the distal disk of the egg processes not smooth (visible only with SEM).

For a more detailed Differential Diagnosis see Supplementary File S1.

### 3.2. Molecular Analysis

**Figure 5:**
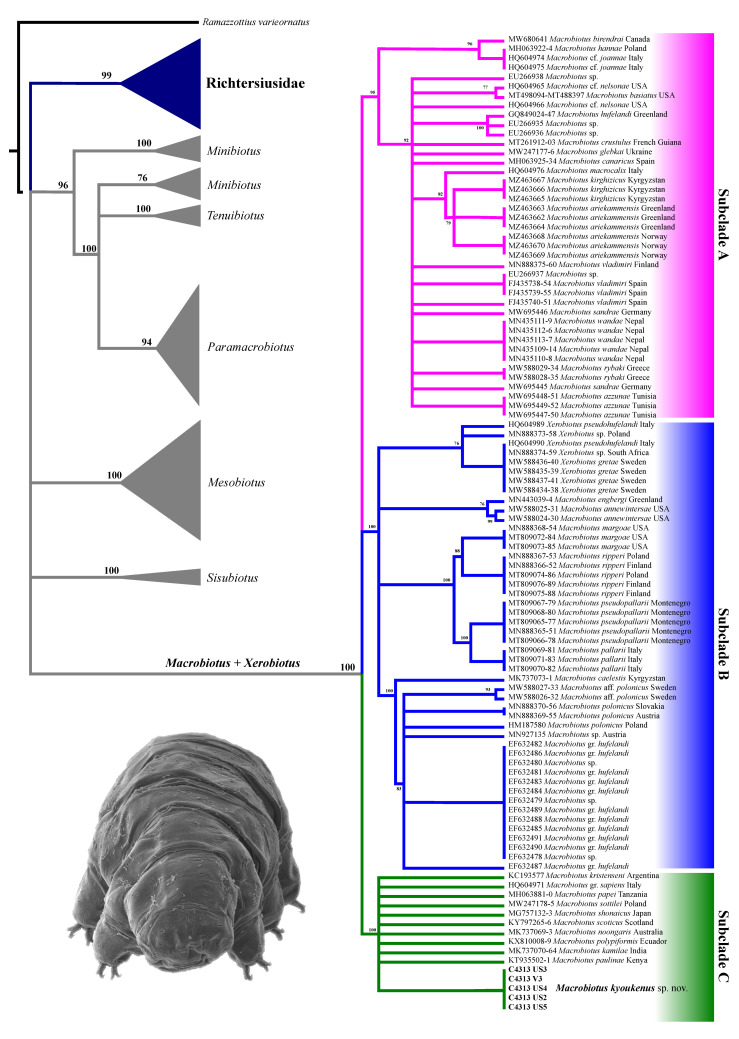
Maximum likelihood phylogenetic tree based on 3659 bp of the 18S and 28S rRNA genes in specimens pertaining to the superfamily Macrobiotoidea. Values in bold denote bootstrap values. All nodes with bootstrap <70% are collapsed. Specimens newly analysed for the present paper are shown in bold; (**lower left**) *In toto* specimen of *M. kyoukenus* sp. nov. (SEM).

The phylogenetic tree computed on 3659 bp of the 18S and 28S genes of all Macrobiotoidea specimens (Figure 5) supports a highly supported phylogenetic line grouping all *Macrobiotus* and *Xerobiotus* specimens. Inside this cluster, three main groups are well supported, corresponding to subclades A, B, and C as identified by Stec et al. [29]. All analysed specimens of *M. kyoukenus* sp. nov. are included in the subclade C.

*M. kyoukenus* sp. nov. specimens are well differentiated from all the other species of the *M. hufelandi* group subclade C (*sensu* [29]), as indicated by the ranges of genetic p-distances:

- ITS-2 (530 bp dataset): 9.6 to 22.7% (Table S3), with the most similar being *M. papei* (MH063921) from Tanzania;
- *cox1* (657 bp dataset): 20.3 to 25.6% (Table S4), with the most similar being *M. shonaicus* from Shizuoka (Japan—LC431585) and *M. papei* (MH057763) from Tanzania.

**Figure 6:**
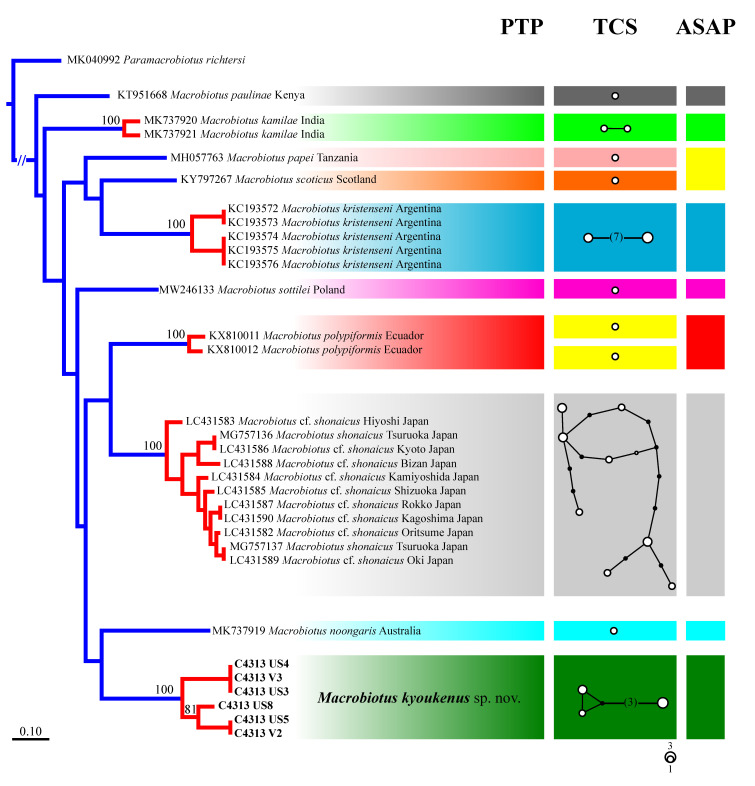
Species delimitation analyses based on 657 bp of the *cox1* gene of *Macrobiotus* species pertaining to the subclade C, *sensu* [29]; (**left**) Tree resulting from the maximum likelihood analysis. Values above branches represent bootstrap values. Results of the Poisson tree process analysis are provided using differently coloured branches: putative species are indicated using transitions from blue-coloured to red-coloured branches. The scale bar shows the number of substitutions per nucleotide position; (**centre**) Haplotype network analysis. Circles denote haplotypes, while circle surface represents haplotype frequency. Black circles show putative/missing haplotypes. Networks falling below the value of the 95% connection limit are disconnected; (**right**) ASAP analysis shows different groups of specimens with the lowest asap-score (2.00) as indicated by rectangles. Discordances between delimitation results are highlighted in yellow. Newly scored haplotypes are in bold.

The *cox1* dataset is the most complete and informative for species delimitation investigation. Therefore, a phylogenetic tree computed with the maximum likelihood method of the *Macrobiotus* specimens pertaining to the subclade C (*sensu* [29]) was utilized for the PTP analysis (Figure 6, left), showing 10 putative species clusters: *M. paulinae*, *M. kamilae*, *M. papei*, *M. scoticus*, *M. kristenseni*, *M. sottilei*, *M. polypiformis*, *M. shonaicus*, *M. noongaris*, and all specimens identified as *M. kyoukenus* sp. nov. This subdivision is validated by both the haplotype network and the ASAP analyses (Figure 6, centre and right, respectively), with minor discordances involving only *M. polypiformis*, *M. papei*, and *M. scoticus* specimens. Present molecular data therefore confirms the validity of the erection of *M. kyoukenus* sp. nov.

### 3.3. Stress Resistance

#### 3.3.1. Desiccation Tolerance

**Figure 7:**
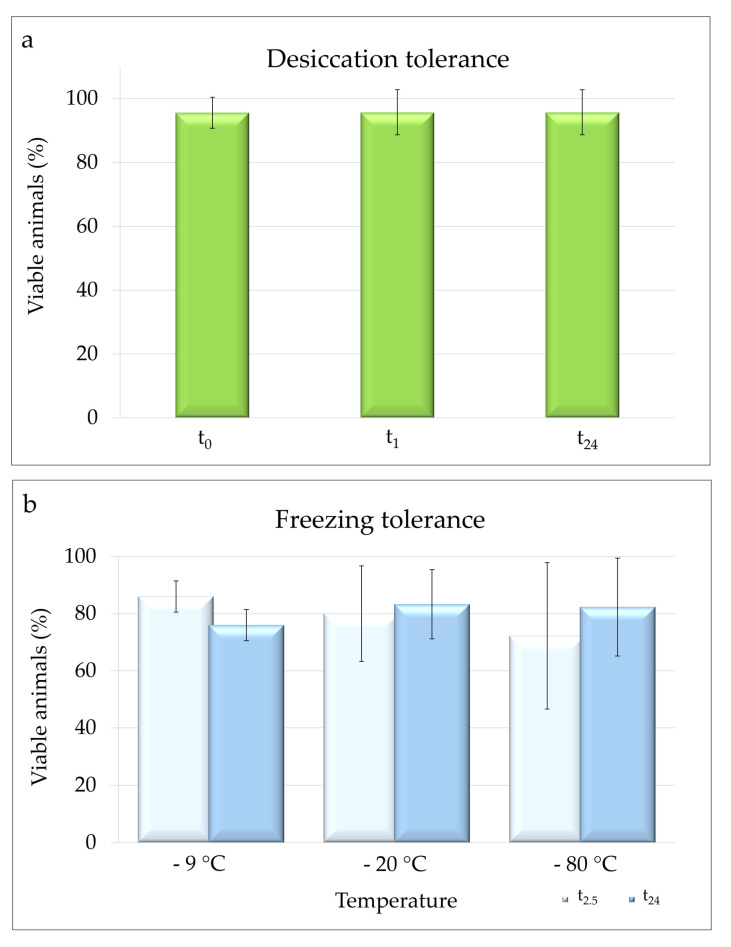
Desiccation and freezing tolerance of *M. kyoukenus* sp. nov. (**a**) Percentage of viable animals immediately after rehydration (t_0_), after 1 h (t_1_) and 24 h (t_24_; final survival); (**b**) Percentage of viable animals at 2.5 h (t_2.5_) post thawing and after 24 h (t_24_; final survival) at different freezing temperature (−9, −20 and −80 °C). Each column represents the mean value of six replicates and the bar on each column represents the standard deviation.

Specimens of *M. kyoukenus* sp. nov. were able to survive desiccation. The mean (± s.d.) percentage of viable animals at t_0_ was 95.58 ± 4.85%, while both at t_1_ and t_24_ (final survival) was 95.71 ± 7.07% (Figure 7a). No significant differences were found among the percentages of viable animals at t_0_, t_1_ and t_24_.

#### 3.3.2. Freezing Tolerance

Specimens of *M. kyoukenus* sp. nov. were able to survive freezing at the three tested temperatures (−9, −20 and −80 °C). The percentages of locomotion performances at t_2.5_ were 86.00 ± 5.48%, 80.00 ± 16.73%, and 72.22 ± 25.64% at −9, −20 or −80 °C, respectively (Figure 7b). The final survivals were 76.00 ± 5.48% at −9 °C, 83.33 ± 12.11% at −20 °C, and 82.33 ± 17.11% at −80 °C (Figure 7b). No significant differences in the percentages of viable animals at t_2.5_ and t_24_ among the three different freezing temperatures were evidenced.

#### 3.3.3. UV Radiation Tolerance

Hydrated and desiccated animals of *M. kyoukenus* sp. nov. tolerate the exposition to both UVB and UVC radiation. The final survivals of the hydrated and desiccated animals used as controls were always of 100%.

**Figure 8:**
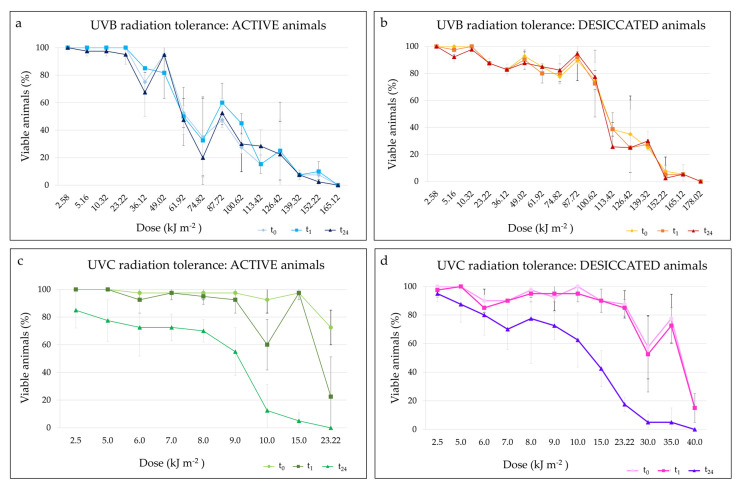
UVB and UVC tolerance of *M. kyoukenus* sp. nov.: percentage of viable animals after exposure to increasing doses of UV radiation; (**a**) Active animals after exposition to UVB radiation. (**b**) Desiccated animals after exposition to UVB radiation; (**c**) Active animals after exposition to UVC radiation; (**d**) Desiccated animals after exposition to UVC radiation. (**a**,**c**) The locomotion performances of animals evaluated immediately after the end of the irradiation (t_0_), after 1 h (t_1_) and 24 h (t_24_) are reported. (**b**,**d**) The locomotion performances of animals evaluated after the end of rehydration (t_0_), after 1 h (t_1_) and 24 h (t_24_) are reported. (**a**–**d**) For each tested UV dose, the mean percentage of viable animals and its standard deviation are shown.

**Figure 9:**
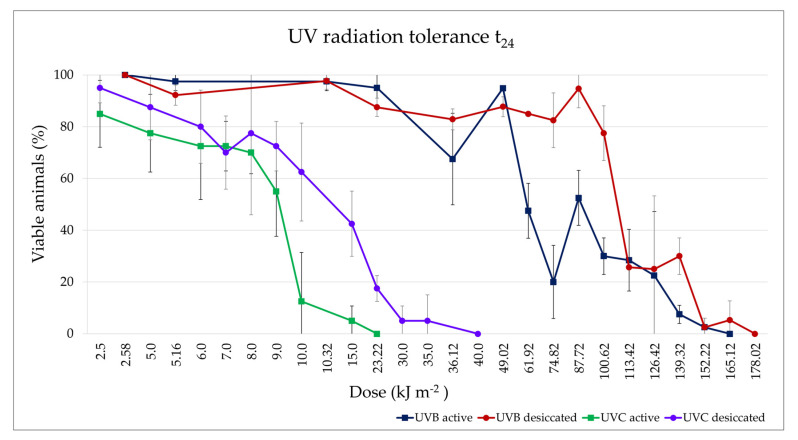
UVB (purple, red) and UVC (green, blue) tolerance of *M. kyoukenus* sp. nov.: final survivals (t_24_) of active and desiccated animals after exposure to increasing doses of UV radiation. For each tested UV dose, the mean percentage of viable animals and its standard deviation are shown.

With regards to UVB irradiation, at each UVB dose, in both hydrated (Figure 8a) and desiccated (Figure 8b) animals, the comparison of percentages of viable animals recorded at t_0_, t_1_ and t_24_ did not show differences. The Spearman correlation test showed a statistically significant decrease in final survival (t_24_; Figure 8a,b and Figure 9) of animals with the increasing in UVB dose in both active (*p* < 0.001) and desiccated specimens (*p* < 0.001). At t_24_, hydrated animals survived up to the UVB dose of 152.22 kJ m^−2^, although the mean final survival was 2.50 ± 3.54% (Figure 8a and Figure 9). On the other hand, desiccated specimens survived up to the UVB dose of 165.12 kJ m^−2^, showing a mean final survival of 5.26 ± 7.44% (Figure 8b and Figure 9). The LD_50_ UVB doses (evaluated at t_24_) were 80.11 kJ m^−2^ and 97.57 kJ m^−2^ in hydrated and desiccated specimens, respectively. Nevertheless, no significant differences were found comparing the trends of viable animals at t_24_ between active and desiccated tardigrades (Figure 8a,b and Figure 9).

Concerning the exposition to UVC radiation, the comparison of percentages of viable animals recorded at t_0_, t_1_ and t_24_ showed significant differences both in hydrated (*p* < 0.001) and desiccated animals (*p* < 0.001). Specifically, no differences were evidenced between the percentages of viable tardigrades at t_0_ and t_1_ both in hydrated and desiccated animals. Moreover, the percentages of the final survivals (t_24_) were significantly lower than those of viable animals at t_0_ and t_1_ both in hydrated (*p* < 0.001) and desiccated (*p* < 0.001) specimens (Figure 8c,d and Figure 9). Even though hydrated animals survived up to the UVC dose of 15.00 kJ m^−2^ with a final survival (t_24_) of 5.00 ± 5.77%, the percentage of viable animals after the same dose of UVC exposition was 97.50 ± 5.00% both at t_0_ and t_1_ (Figure 8c and Figure 9). Desiccated tardigrades survived up to the UVC dose of 35.00 kJ m^−2^ showing a final survival of 5.00 ± 10.00%, lower than the viabilities at t_0_ (77.50 ± 17.08%) and t_1_ (72.50 ± 12.58%; Figure 8b and Figure 9). In addition, the final survivals (t_24_) significantly decrease with the increase in UVC doses in both active (*p* < 0.001) and desiccated (*p* < 0.001) tardigrades (Figure 8c,d and Figure 9). The LD_50_ UVC doses calculated at t_24_ were 8.44 kJ m^−2^ in the active specimens and 14.42 kJ m^−2^ in the desiccated ones. However, no statistical differences were recorded between the trends of the animal final survivals (t_24_) comparing hydrated and desiccated specimens.

Comparing the trends of the final survivals at t_24_ of animals exposed to UVB and UVC radiation, significant differences were evidenced (*p* < 0.001; Figure 9). In particular, the final survivals of hydrated and desiccated animals exposed to UVC radiation were significantly lower respect to those of animals exposed to UVB doses (*p* < 0.001; Figure 9).

## 4. Discussion

The integrated morphological and molecular data clearly show that the tardigrade specimens collected in Tsukuba (Japan) belong to the *Macrobiotus hufelandi* group (Figure 1, Figure 2, Figure 3, Figure 4 and Figure 5). Phylogenetic data point out that all analysed specimens belong to the subclade C *sensu* [29] nested inside a *Macrobiotus* + *Xerobiotus* (Figure 5). The systematic and taxonomic status and relationships of *Macrobiotus* and *Xerobiotus* is recently under debate [29,33,55]. Moreover, subclade C does not show clear synapomorphies. Its species have the first macroplacoid with a less evident constriction (but this character is shared with species pertaining to subclade A); the transversal dorsal crests in the buccal pharyngeal apparatus are overlapping and may appear as fused in the LM (but this is not true for *M. kristenseni* [28]). Therefore, the various evolutionary lineages that are found within the *Macrobiotus* cluster using the molecular approach should be analysed more thoroughly, with the scope of pointing out morphological synapomorphies. Only when this integrated approach will be applied, new supraspecific taxa can be established.

Moreover, morphological data and species delimitation analysis showed quite clear results, pointing out to that all analysed tardigrade specimens from Tsukuba belong to a new species, *Macrobiotus kyoukenus* sp. nov. (Figure 1, Figure 2, Figure 3, Figure 4 and Figure 6). This is only the third finding in Japan of a species pertaining to the *Macrobiotus hufelandi* group, which is quite a surprising result, considering that this species group is one of the most speciose and widespread. Present data therefore increases the number of water bear species in Japan, contributing to discover its tardigrade biodiversity.

The morphological analysis on *Macrobiotus kyoukenus* sp. nov. also showed the presence of different cuticular sensory areas. Little is known about the sensory organs in eutardigrades. In the order Apochela, the “circumoral sensory field” (COS) (a.k.a. peribuccal sense organ) and the “antero-lateral sensory field” (ALS) are characterized by papillae, while in Parachela, they are difficult to detect because they are not associated with cuticle eversions [56,57,58,59,60]. The observation of some peculiar characteristics of the superficial cuticle in correspondence of sensory areas allows detecting the sensory fields in Parachela. The presence of areas with very small (≤ 0.2 µm) cuticular pores (see e.g., [60,61,62,63], Figure 3C–E) are associated to ALS and the “postero-lateral sensory field” (PLS), while COS is characterized by a different cuticular pattern with respect to the surrounding area (Figure 2C). In *Macrobiotus kyoukenus* sp. nov., the COS (corresponding to the smooth cuticular ring around the mouth opening; Figure 2C), ALS (corresponding to the small areas with very small pores lateral to the mouth opening; Figure 3C), and PLS (corresponding to the small areas with very small pores present on the dorso-lateral sides of the head, Figure 3D,E) were identified. All these three areas were found also in other Parachela belonging to *Ramazzottius* and *Cryoconicus* [60]. In *Macrobiotus kyoukenus* sp. nov., two further potential sensory areas were discovered. One is associated with the cloaca; specifically, two small areas are present on the dorsal side of the cloacal opening characterized by several small pores (Figure 3F). Based on its position, it can be defined as a “cloaca sensory field” (CLS), and it could be related to reproduction (e.g., gamete release and/or copulation). The second new possible sensory field is associated with the flat bulge on the hind legs, which shows at least three very small pores (Figure 3A). Interestingly, a bulge on the external side of the hind legs is also found in species of other genera (e.g., *Ramazzottius* and *Macrobiotus*). Therefore, it would be interesting in the future to confirm the presence of this sensory field in other parachelan species.

*Macrobiotus kyoukenus* sp. nov. tolerates both desiccation and freezing entering in cryptobiosis (anhydrobiosis and cryobiosis, respectively). The high survival of this species to desiccation is in line with the anhydrobiotic performances showed by other species of tardigrades colonizing terrestrial habitats, such as lichens, leaf litter and mosses [2,4,6,64,65]. In addition, the obtained data on the studied species further confirm the higher capability to tolerate freezing of terrestrial tardigrades with respect to freshwater ones [54,66].

Ultraviolet irradiation can be divided into three types based on the wavelength range: UVA from 315 to 400 nm, UVB from 280 to 315 nm, and UVC from 100 to 280 nm [67]. Among these types of UV radiation, UVC is the most energetic and therefore dangerous. Fortunately, the atmosphere and ozone layer can filter out UVC [67]. UVC and UVB are directly absorbed by tardigrade DNA, damaging it [68]. Some organisms are supposed to possess special mechanisms to mitigate DNA damages due to irradiation, as they show an extraordinary tolerance against radiation [69]. Among these organisms, tardigrades are able to tolerate irradiation both in an active and desiccated state [9,10,12,68,70]. Similarly, *M. kyoukenus* sp. nov. tolerates UVB and UVC both in an active and desiccated state. At variance with previous reports where active and desiccated tardigrades evidenced different abilities to withstand UV radiation [9,10], in this study active and dried specimens show similar level of resistance. Moreover, among the tardigrade species studied so far, *M. kyoukenus* sp. nov. is the most resistant to ultraviolet radiation, as it tolerates doses of 165.12 kJ m^−2^ and 35.00 kJ m^−2^ of UVB and UVC, respectively. In particular, analysed specimens are more resistant and resilient to UVB then to UVC. This higher sensitivity of *M*. *kyoukenus* sp. nov. specimens to UVC doses with respect to UVB is a consequence of the high impact on survival of the most energetic and dangerous UVC radiation. After the exposition to UVC, animals are not able to repair damages as evidenced by the higher motilities of animals at t_0_ and t_1_ with respect to the motilities recorded at the end of the experiments (t_24_). Among DNA damages, a strong dose-dependent increase in dimer formation after UVC irradiation was evidenced in tardigrades [10]. UVC irradiation on embryos of the model organism zebrafish caused a rapid decrease in survival at a dose of 0.075 kJ m^−2^ [67]. Considering that the survival rates of the irradiated embryos at 0.045 kJ m^−2^ were 100%, this intensity might represent the limit to which the embryo’s internal biodefense system can protect against UVC radiation [67]. Similarly, tardigrades can withstand to UVC up to of 35.00 kJ m^−2^ thanks to the internal biodefense system, even though a consistent drop in survival was recorded around the exposure dose of 10.0 kJ m^−2^. Since the atmosphere filters out UVC radiation [67] and the annual dose of UV recorded in 2018 at Tsukuba is about 700 kJ m^−2^ [71], in nature the animals belonging to the tested population are not exposed to the high experimental tested doses. However, the ongoing climate changes and the ozone depletion will lead to an increase in UV irradiation on the Earth, and consequently of the selective pressure, but obtained data suggest that *M. kyoukenus* sp. nov. could be able to easily adapt to the new possible extreme environmental conditions. 

The stress tolerance capabilities of *M. kyoukenus* sp. nov to different environmental conditions (desiccation, rapid freezing, and high levels of ultraviolet radiations) show that this new species could become a new emerging model for stress resistance studies.

## Figures and Tables

**Table 1:** List of tardigrade specimens utilized for molecular analyses. N/A = not available.

**Specimen**	**Voucher** **Specimen Type**	**18S**	**28S**	**ITS-2**	***cox1***
C4313 V2	hologenophore	N/A	ON818306	N/A	ON809460
C4313 V3	hologenophore	ON818312	ON818307	ON818300	ON809461
C4313 US2	hologenophore	ON818313	ON818308	N/A	N/A
C4313 US3	hologenophore	ON818314	ON818309	ON818301	ON809462
C4313 US4	hologenophore	ON818315	ON818310	ON818302	ON809463
C4313 US5	hologenophore	ON818316	ON818311	ON818303	ON809464
C4313 US6	hologenophore	N/A	N/A	ON818304	N/A
C4313 US7	hologenophore	N/A	N/A	ON818305	N/A
C4313 US8	hologenophore	N/A	N/A	N/A	ON809465

## Data Availability

Data presented in this study are included within the article and its additional files. The raw data used and/or analysed during the present study are available from the corresponding author on reasonable request.

## Supplementary Materials

The following are available online at https://www.mdpi.com/article/10.3390/insects13070634/s1, Table S1: Morphometric measurements; Table S2: GenBank Macrobiotoidea sequences utilised for comparison; Supplementary File S1: Extended differential diagnosis; Table S3: *cox1* gene pairwise p-distances; Table S4: ITS-2 gene pairwise p-distances.

## References

[1] Guidetti R., Altiero T., Rebecchi L. (2011). On dormancy strategies in tardigrades. J. Insect Physiol..

[2] Rebecchi L., Altiero T., Guidetti R. (2007). Anhydrobiosis: The extreme limit of desiccation tolerance. ISJ Invert. Surviv. J..

[3] Møbjerg N., Halberg K.A., Jørgensen A., Persson D., Bjørn M., Ramløv H., Kristensen R.M. (2011). Survival in extreme environments—On the current knowledge of adaptations in tardigrades. Acta Physiol..

[4] Horikawa D.D., Kunieda T., Abe W., Watanabe M., Nakahara Y., Yukuhiro F., Sakashita T., Hamada N., Wada S., Funayama T. (2008). Establishment of a rearing system of the extremotolerant tardigrade *Ramazzottius varieornatus*: A new model animal for astrobiology. Astrobiology.

[5] Jönsson K.I., Rabbow E., Schill R.O., Harms-Ringdahl M., Rettberg P. (2008). Tardigrades survive exposure to space in low Earth orbit. Curr. Biol..

[6] Rebecchi L., Cesari M., Altiero T., Frigieri A., Guidetti R. (2009). Survival and DNA degradation in anhydrobiotic tardigrades. J. Exp. Biol..

[7] Neves R.C., Hvidepil L.K.B., Sørensen-Hygum T.L., Stuart R.M., Møbjerg N. (2020). Thermotolerance experiments on active and desiccated states of *Ramazzottius varieornatus* emphasize that tardigrades are sensitive to high temperatures. Sci. Rep. UK.

[8] Møbjerg N., Neves R.C. (2021). New insights into survival strategies of tardigrades. Comp. Biochem. Phys. A.

[9] Altiero T., Guidetti R., Caselli V., Cesari M., Rebecchi L. (2011). Ultraviolet radiation tolerance in hydrated and desiccated eutardigrades. J. Zool. Syst. Evol. Res..

[10] Horikawa D.D., Cumbers J., Sakakibara I., Rogo D., Leuko S., Harnoto R., Arakawa K., Katayama T., Kunieda T., Toyoda A. (2013). Analysis of DNA repair and protection in the Tardigrade *Ramazzottius varieornatus* and *Hypsibius dujardini* after exposure to UVC radiation. PLoS ONE.

[11] Hashimoto T., Horikawa D.D., Saito Y., Kuwahara H., Kozuka-Hata H., Shin I.T., Minakuchi Y., Ohishi K., Motoyama A., Aizu T. (2016). Extremotolerant tardigrade genome and improved radiotolerance of human cultured cells by tardigrade-unique protein. Nat. Commun..

[12] Giovannini I., Altiero T., Guidetti R., Rebecchi L. (2018). Will the Antarctic tardigrade *Acutuncus antarcticus* be able to withstand environmental stresses related to global climate change?. J. Exp. Biol..

[13] Jönsson K.I. (2019). Radiation tolerance in tardigrades: Current knowledge and potential applications in medicine. Cancers.

[14] Guidetti R., Bertolani R. (2005). Tardigrade taxonomy: An updated checklist of the taxa and a list of characters for their identification. Zootaxa.

[15] Degma P., Guidetti R. (2007). Notes to the current checklist of Tardigrada. Zootaxa.

[16] Mathews G. (1937). Tardigrada from Japan. Peking Nat. Hist. Bull..

[17] Utsugi K. (1986). Urban tardigrades in Kyushu. Zool. Sci..

[18] Utsugi K. (1988). Tardigrades in Hokkaido area. Zool. Sci..

[19] Hatai S. (1956). On the Japanese Tardigrada. Sci. Rep. Yokosuka City Mus..

[20] Utsugi K. (1996). Study on terrestrial tardigrades in Japan. II. Summary of urban tardigrades in Japan. Nat. Environ. Sci. Res..

[21] Suzuki A.C., Heard L., Sugiura K. (2018). Terrestrial tardigrades from Mikurajima Island (the first report). Mikurensis.

[22] Utsugi K. (1987). Urban tardigrades in Hokuriku area. Zool. Sci..

[23] Biserov V.I., Dudichev A.L., Biserova N.M. (2001). Preliminary data on tardigrades of Lake Biwa (Japan). Arthropoda Sel..

[24] Ishida M., Matsui T. (2007). Noteworthy tardigrades from Kochi Prefecture. Nat. Environ. Sci. Res..

[25] Stec D., Arakawa K., Michalczyk Ł. (2018). An integrative description of *Macrobiotus shonaicus* sp. nov. (Tardigrada: Macrobiotidae) from Japan with notes on its phylogenetic position within the *hufelandi* group. PLoS ONE.

[26] Sugiura K., Arakawa K., Matsumoto M. (2020). Distribution of *Macrobiotus shonaicus* Stec, Arakawa & Michalczyk, 2018 (Tardigrada: Eutardigrada: Macrobiotidae) in Japan. Zootaxa.

[27] Bertolani R., Rebecchi L. (1993). A revision of the *Macrobiotus hufelandi* group (Tardigrada, Macrobiotidae), with some observations on the taxonomic characters of eutardigrades. Zool. Scr..

[28] Guidetti R., Peluffo J.R., Rocha A.M., Cesari M., Moly de Peluffo M.C. (2013). The morphological and molecular analyses of a new South American urban tardigrade offer new insights on the biological meaning of the *Macrobiotus hufelandi* group of species (Tardigrada: Macrobiotidae). J. Nat. Hist..

[29] Stec D., Vecchi M., Calhim S., Michalczyk Ł. (2021). New multilocus phylogeny reorganises the family Macrobiotidae (Eutardigrada) and unveils complex morphological evolution of the *Macrobiotus hufelandi* group. Mol. Phylogenet. Evol..

[30] Bertolani R., Biserov V., Rebecchi L., Cesari M. (2011). Taxonomy and biogeography of tardigrades using an integrated approach: New results on species of the *Macrobiotus hufelandi* group. Invertebr. Zool..

[31] Kaczmarek Ł., Michalczyk Ł. (2017). The *Macrobiotus hufelandi* group (Tardigrada) revisited. Zootaxa.

[32] Michalczyk Ł., Kaczmarek Ł. (2013). The Tardigrada Register: A comprehensive online data repository for tardigrade taxonomy. J. Limnol..

[33] Massa E., Guidetti R., Cesari M., Rebecchi L., Jönsson K.I. (2021). Tardigrades of Kristianstads Vattenrike Biosphere Reserve with description of four new species from Sweden. Sci. Rep..

[34] Bartels P.J., Nelson D.R., Exline R.P. (2011). Allometry and the removal of body size effects in the morphometric analysis of tardigrades. J. Zool. Syst. Evol. Res..

[35] Bertolani R., Guidetti R., Marchioro T., Altiero T., Rebecchi L., Cesari M. (2014). Phylogeny of Eutardigrada: New molecular data and their morphological support lead to the identification of new evolutionary lineages. Mol. Phylogenet. Evol..

[36] Cesari M., Vecchi M., Palmer A., Bertolani R., Pilato G., Rebecchi L., Guidetti R. (2016). What if the claws are reduced? Morphological and molecular phylogenetic relationships of the genus *Haplomacrobiotus* May, 1948 (Eutardigrada, Parachela). Zool. J. Linn. Soc..

[37] Stec D., Morek W., Gąsiorek P., Michalczyk Ł. (2018). Unmasking hidden species diversity within the *Ramazzottius oberhaeuseri* complex, with an integrative redescription of the nominal species for the family Ramazzottiidae (Tardigrada: Eutardigrada: Parachela). Syst. Biodivers..

[38] Bertolani R., Rebecchi L., Giovannini I., Cesari M. (2011). DNA barcoding and integrative taxonomy of *Macrobiotus hufelandi* C.A.S. Schultze 1834, the first tardigrade species to be described, and some related species. Zootaxa.

[39] Katoh K., Misawa K., Kuma K.I., Miyata T. (2002). MAFFT: A novel method for rapid multiple sequence alignment based on fast Fourier transform. Nucleic Acids Res..

[40] Katoh K., Rozewicki J., Yamada K.D. (2017). MAFFT online service: Multiple sequence alignment, interactive sequence choice and visualization. Brief. Bioinform..

[41] Stamatakis A. (2014). RAxML version 8: A tool for phylogenetic analysis and post-analysis of large phylogenies. Bioinformatics.

[42] Guindon S., Gascuel O. (2003). A simple, fast and accurate method to estimate large phylogenies by maximum-likelihood. Syst. Biol..

[43] Darriba D., Taboada G.L., Doallo R., Posada D. (2012). jModelTest 2: More models, new heuristics and parallel computing. Nat. Methods.

[44] Yang Z. (2014). Molecular Evolution: A Statistical Approach.

[45] Stamatakis A., Hoover P., Rougemont J. (2008). A rapid bootstrap algorithm for the RAxML web servers. Syst. Biol..

[46] Kumar S., Stecher G., Li M., Knyaz C., Tamura K. (2018). MEGA X: Molecular evolutionary genetics analysis across computing platforms. Mol. Biol. Evol..

[47] Templeton A.R., Crandall K.A., Sing C.F. (1992). A cladistic analysis of phenotypic association with haplotypes inferred from restriction endonuclease mapping and DNA sequence data. III. Cladogram estimation. Genetics.

[48] Clement M., Posada D., Crandall K. (2000). TCS: A computer program to estimate gene genealogies. Mol. Ecol..

[49] Santos A.M., Cabezas M.P., Tavares A.I., Xavier R., Branco M. (2016). tcsBU: A tool to extend TCS network layout and visualization. Bioinformatics.

[50] Hart M.W., Sunday J. (2007). Things fall apart: Biological species form unconnected parsimony networks. Biol. Lett..

[51] Puillandre N., Brouillet S., Achaz G. (2021). ASAP: Assemble species by automatic partitioning. Mol. Ecol. Resour..

[52] Zhang J., Kapli P., Pavlidis P., Stamatakis A. (2013). A general species delimitation method with applications to phylogenetic placements. Bioinformatics.

[53] Rebecchi L., Altiero T., Guidetti R., Cesari M., Bertolani R., Negroni M., Rizzo A.M. (2009). Tardigrade resistance to space effects: First results of experiments on the LIFE-TARSE mission on FOTON-M3 (September 2007). Astrobiology.

[54] Guidetti R., Altiero T., Bertolani R., Grazioso P., Rebecchi L. (2011). Survival of freezing by hydrated tardigrades inhabiting terrestrial and freshwater habitats. Zoology.

[55] Stec D., Vončina K., Kristensen R.M., Michalczyk Ł. (2022). The *Macrobiotus ariekammensis* species complex provides evidence for parallel evolution of claw elongation in macrobiotid tardigrades. Zool. J. Linn. Soc..

[56] Walz B. (1978). Electron microscopic investigation of cephalic sense organs of the tardigrade *Macrobiotus hufelandi* C.A.S. Schulze. Zoomorphologie.

[57] Wiederhöft H., Greven H. (1996). The cerebral ganglia of *Milnesium tardigradum* Doyère (Apochela, Tardigrada): Three dimensional reconstruction and notes on their ultrastructure. Zool. J. Linn. Soc..

[58] Wiederhöft H., Greven H. (1999). Notes on head sensory organs of *Milnesium tardigradum* Doyère, 1840 (Apochela, Eutardigrada). Zoologischer Anzeiger.

[59] Biserova N.M., Kuznetsova K.G. (2012). Head sensory organs of *Halobiotus stenostomus* (Eutardigrada, Hypsibiidae). Biol. Bull..

[60] Guidetti R., Cesari M., Giovannini I., Ebel C., Förschler M.I., Rebecchi L., Schill R.O. (2022). Morphology and taxonomy of the genus *Ramazzottius* (Eutardigrada; Ramazzottidae) with the integrative description of *Ramazzottius kretschmanni* sp. nov. Eur. Zool. J..

[61] Dastych H. (2011). *Ramazzottius agannae* sp. nov., a new tardigrade species from the nival zone of the Austrian Central Alps (Tardigrada). Ent. Mitt. Zool. Mus. Hambg..

[62] Kaczmarek L., Michalczyk L., Diduszko D. (2006). *Ramazzottius bunikowskae*, a new species of Tardigrada (Eutardigrada, Hypsibiidae) from Russia. Zootaxa.

[63] Tumanov D.V. (2020). Integrative description of *Mesobiotus anastasiae* sp. nov. (Eutardigrada, Macrobiotoidea) and first record of *Lobohalacarus* (Chelicerata, Trombidiformes) from the Republic of South Africa. Eur. J. Taxon..

[64] Jönsson K.I., Borsari S., Rebecchi L. (2001). Anhydrobiotic survival in populations of the tardigrades *Richtersius coronifer* and *Ramazzottius oberhaeuseri* from Italy and Sweden. Zool. Anz..

[65] Horikawa D.D., Higashi S. (2004). Desiccation tolerance of the tardigrade *Milnesium tardigradum* collected in Sapporo, Japan, and Bogor, Indonesia. Zool. Sci..

[66] Hengherr S., Worland M.R., Reuner A., Brümmer F., Schill R.O. (2009). Freeze tolerance, supercooling points and ice formation: Comparative studies on the subzero temperature survival of limno-terrestrial tardigrades. J. Exp. Biol..

[67] Zang L., Shimada Y., Miyake H., Nishimura N. (2022). Transcriptome analysis of molecular response to UVC irradiation in zebrafish embryos. Ecotox. Environ. Saf..

[68] Jönsson K.I., Levine E.B., Wojcik A., Haghdoost S., Harms-Ringdahl M., Schill R.O. (2018). Environmental adaptations: Radiation tolerance. Water Bears: The Biology of Tardigrades.

[69] Hashimoto T., Kunieda T. (2017). DNA protection protein, a novel mechanism of radiation tolerance: Lessons from Tardigrades. Life.

[70] May R.M., Maria M., Guimard J. (1964). Actions différentielles des rayons X et Ultraviolets sur le tardigrade *Macrobiotus areolatus*, à l´état actif et desséché. Bull. Biol. Fr. Belg..

[71] United Nations Environment Programme (2018). Environmental Effects and Interactions of Stratospheric Ozone Depletion, UV Radiation, and Climate Change: 2018 Assessment Report.

