# Supplementary material for: Resistance to Extreme Stresses by a Newly Discovered Japanese Tardigrade Species, Macrobiotus kyoukenus (Eutardigrada, Macrobiotidae)
Source: Insects. 2022 Jul 15;13(7):634. doi: 10.3390/insects13070634 (PMC9315711; doi:10.3390/insects13070634)
Supplement: Supplementary file 1 [file insects-13-00634-s001.zip › Cesari et al Supplementary File S3.pdf]

Supplementary File S3

Differential diagnosis

Macrobiotus kyoukenus n. sp. differs from the most similar species for the following characters:

Macrobiotus canaricus Stec, Krzywański & Michalczyk, 2018 by a smaller size of the meshes (pores) present on the egg surface between processes, the more regular margin of the distal disk of the egg processes (serrated in *M. canaricus*), and a more irregular surface (visible only with SEM) of the distal disk of the egg processes (smooth with few microgranules in *M. canaricus*);

Macrobiotus dulciporus Roszkowska, Gawlak, Draga & Kaczmarek, 2019, by absence of large lenticular pores (up to 5 µm) on cuticle surface of animals, less evident patch of granules on the external side of the legs, dorsal transversal crests of the buccal armature in contact with each other (with LM observation), presence of meshes (pores) on the egg surface between processes, the more regular margin of the distal disk of the egg processes (serrated in *M. dulciporus*), more irregular surface (visible only with SEM) of the distal disk of the egg processes (smooth in *M. dulciporus*);

Macrobiotus engbergi Stec, Tumanov & Kristensen, 2020, by absence of large lenticular pores (up to 8 µm) on cuticle surface of animals, dorsal transversal crests of the buccal armature in contact with each other (with LM observation), presence of meshes (pores) on the egg surface between processes, the more regular margin of the distal disk of the egg processes (serrated in *M. engbergi*);

Macrobiotus hannae Nowak & Stec, 2018, by dorsal transversal crests of the buccal armature in contact with each other (with LM observation), smooth lunules in the hind legs, the more regular margin of the distal disk of the egg processes (serrated in *M. hannae*), more irregular surface (visible only with SEM) of the distal disk of the egg processes (smooth with several microgranules in *M. hannae*);

Macrobiotus humilis Binda & Pilato, 2001, by the smaller size of the meshes (pores) present on the egg surface between processes, egg processes with a larger diameter of the distal disk respect to the basal diameter, the more regular margin of the distal disk of the egg processes (serrated in *M. humilis*);

Macrobiotus kamilae Coughlan & Stec, 2019, by smooth lunules in the hind legs, egg processes with a larger diameter of the distal disk respect to the basal diameter, more irregular surface (visible only with SEM) of the distal disk of the egg processes (smooth in *M. kamilae*);

Macrobiotus nebrodensis Pilato, Sabella, D'Urso & Lisi, 2017, by smaller size of meshes (pores) present on egg surface between processes, the presence of an uniform shape of egg processes (irregular in *M. nebrodensis*);

Macrobiotus noogaris Coughlan & Stec, 2019, by smooth lunules in the hind legs, and egg processes with a larger diameter of the distal disk respect to the basal diameter;

Macrobiotus papei Stec, Kristensen & Michalczyk, 2018, by smooth lunules in the hind legs and terminal disc of the egg processes without thin filaments;

Macrobiotus sandrae Bertolani & Rebecchi, 1993, by dorsal transversal crests of the buccal armature in contact with each other (with LM observation), smooth lunules in the hind legs, and smaller size of meshes (pores) present on egg surface between processes;

Macrobiotus sottilei Pilato, Kiosya, Lisi & Sabella, 2012, by smooth lunules in the hind legs and egg, and egg processes with a larger diameter of the distal disk respect to the basal diameter.
