# Supplementary material for: Resistance to Extreme Stresses by a Newly Discovered Japanese Tardigrade Species, Macrobiotus kyoukenus (Eutardigrada, Macrobiotidae)
Source: Insects. 2022 Jul 15;13(7):634. doi: 10.3390/insects13070634 (PMC9315711; doi:10.3390/insects13070634)
Supplement: Supplementary file 1 [file insects-13-00634-s001.zip › Cesari et al Table S1.pdf]

Table S1. Measurements (in µm) and *pt* values of morphological structures of the holotype and paratypes of *Macrobiotus kyoukenus* sp. nov.

CHARACTER	N	RANGE						MEAN		SD		Holotype		Thorpe's Normalization		
		µm			*pt*			µm	*pt*	µm	*pt*	µm	*pt*	*b*	*a**	*p_value*
Body length	30	214	–	535	*748*	–	*1183*	362	*934*	79	*105*	363	*924*	*1.4335*	*90.2592*	*0.4738*
Buccopharyngeal tube																
Buccal tube length	30	28.5	–	48.1		–		38.5	–	5.6	–	39.2	–	*1.0000*	*0.0000*	*1.0000*
Stylet support insertion point	30	21.5	–	37.8	*70.4*	–	*79.4*	29.2	*75.8*	4.5	*2.0*	29.3	*74.8*	*1.0624*	*-2.9876*	*0.9606*
Buccal tube external width	30	3.8	–	7.4	*12.4*	–	*16.4*	5.6	*14.4*	1.1	*1.0*	6.0	*15.2*	*1.3046*	*-2.6166*	*0.7416*
Buccal tube internal width	30	2.5	–	5.2	*8.6*	–	*11.6*	3.9	*10.1*	0.9	*1.0*	4.5	*11.6*	*1.4894*	*-2.5604*	*0.6617*
Placoid lengths																
Macroplacoid 1	30	7.2	–	14.3	*22.3*	–	*31.7*	10.3	*26.8*	2.1	*2.6*	10.3	*26.3*	*1.1868*	*-1.6845*	*0.6733*
Macroplacoid 2	30	4.1	–	8.3	*13.3*	–	*17.8*	5.9	*15.3*	1.3	*1.3*	5.7	*14.5*	*1.3478*	*-2.8446*	*0.6579*
Microplacoid	30	1.8	–	3.9	*5.0*	–	*8.8*	2.5	*6.5*	0.6	*0.9*	3.1	*7.9*	*1.0167*	*0.7512*	*0.9399*
Macroplacoid row	30	12.3	–	22.8	*40.0*	–	*50.3*	17.3	*45.0*	3.2	*3.1*	16.9	*43.1*	*1.1409*	*-2.7832*	*0.8176*
Placoid row	30	14.7	–	27.3	*47.1*	–	*59.7*	20.5	*53.2*	3.6	*3.4*	20.9	*53.4*	*1.1066*	*-1.7757*	*0.8487*
Claw 1 heights																
External primary branch	30	7.3	–	11.1	*21.9*	–	*28.9*	9.5	*24.8*	1.1	*1.5*	10.2	*25.9*	*0.7394*	*3.2172*	*0.4294*
External secondary branch	30	6.1	–	9.5	*15.1*	–	*22.1*	7.3	*19.1*	0.9	*1.9*	7.2	*18.4*	*0.5978*	*2.4741*	*0.0038*
Claw 2-3 heights																
External primary branch	30	8.1	–	11.7	*23.6*	–	*31.6*	10.2	*26.8*	0.9	*2.1*	10.3	*26.3*	*0.5920*	*4.6595*	*0.0997*
External secondary branch	30	6.4	–	9.8	*17.3*	–	*23.3*	7.6	*20.0*	0.7	*1.9*	7.9	*20.1*	*0.4927*	*3.9120*	*0.0001*
Claw 4 lengths																
Posterior primary branch	30	8.8	–	13.8	*27.4*	–	*37.4*	12.1	*31.5*	1.4	*2.3*	12.4	*31.5*	*0.7172*	*4.2409*	*0.2308*
Posterior secondary branch	30	6.4	–	11.1	*19.2*	–	*28.1*	8.8	*23.1*	1.2	*2.3*	8.2	*20.9*	*0.7742*	*2.9594*	*0.2376*
