# Supplementary material for: Resistance to Extreme Stresses by a Newly Discovered Japanese Tardigrade Species, Macrobiotus kyoukenus (Eutardigrada, Macrobiotidae)
Source: Insects. 2022 Jul 15;13(7):634. doi: 10.3390/insects13070634 (PMC9315711; doi:10.3390/insects13070634)
Supplement: Supplementary file 1 [file insects-13-00634-s001.zip › Cesari et al Table S2.pdf]

Table S2. Macrobiotoidea sequences from GenBank utilised for comparison.

Species	GenBank Accession Number			
	**18S**	**28S**	**ITS-2**	***cox1***
Ramazzottius varieornatus	BDGG01000030	BDGG01000030		
Macrobiotus hufelandi Greenland	GQ849024	GQ849047		
Macrobiotus vladimiri Spain	FJ435738	FJ435754		
Macrobiotus vladimiri Spain	FJ435739	FJ435755		
Macrobiotus vladimiri Spain	FJ435740	FJ435751		
Macrobiotus vladimiri Finland	MN888375	MN888360		
Macrobiotus cf. *nelsonae* USA	HQ604965			
Macrobiotus cf. *nelsonae* USA	HQ604966			
Macrobiotus gr. *sapiens* Italy	HQ604971			
Macrobiotus sapiens Croatia			GQ403680	
Macrobiotus cf. *joannae* Italy	HQ604974			
Macrobiotus cf. *joannae* Italy	HQ604975			
Macrobiotus macrocalix Italy	HQ604976			
Macrobiotus polonicus Poland	HM187580			
Macrobiotus polonicus Austria	MN888369	MN888355		
Macrobiotus polonicus Slovakia	MN888370	MN888356		
Macrobiotus aff. *polonicus* Sweden	MW588026	MW588032		
Macrobiotus aff. *polonicus* Sweden	MW588027	MW588033		
Macrobiotus annewintersae USA	MW588024	MW588030		
Macrobiotus annewintersae USA	MW588025	MW588031		
Macrobiotus engbergi Greenland	MN443039	MN443034		
Macrobiotus caelestis Kyrgyzstan	MK737073	MK737071		
Macrobiotus pseudopallarii Montenegro	MN888365	MN888351		
Macrobiotus pseudopallarii Montenegro	MT809065	MT809077		
Macrobiotus pseudopallarii Montenegro	MT809066	MT809078		
Macrobiotus pseudopallarii Montenegro	MT809067	MT809079		
Macrobiotus pseudopallarii Montenegro	MT809068	MT809080		
Macrobiotus pallarii Italy	MT809069	MT809081		
Macrobiotus pallarii Italy	MT809070	MT809082		
Macrobiotus pallarii Italy	MT809071	MT809083		
Macrobiotus paulinae Kenya	KT935502	KT935501	KT935500	KT951668
Macrobiotus polypiformis Ecuador	KX810008	KX810009	KX810010	KX810011
Macrobiotus polypiformis Ecuador				KX810012
Macrobiotus kristenseni Argentina	KC193577			KC193572
Macrobiotus kristenseni Argentina				KC193573
Macrobiotus kristenseni Argentina				KC193574
Macrobiotus kristenseni Argentina				KC193575
Macrobiotus kristenseni Argentina				KC193576
Macrobiotus scoticus Scotland	KY797265	KY797266	KY797268	KY797267
Macrobiotus shonaicus Japan	MG757132	MG757133	MG757134	MG757136
Macrobiotus shonaicus Japan			MG757135	MG757137
Macrobiotus shonaicus Japan			LC431591	LC431582
Macrobiotus shonaicus Japan			LC431592	LC431583
Macrobiotus shonaicus Japan			LC431593	LC431584
Macrobiotus shonaicus Japan			LC431594	LC431585
Macrobiotus shonaicus Japan			LC431595	LC431586
Macrobiotus shonaicus Japan			LC431596	LC431587
Macrobiotus shonaicus Japan			LC431597	LC431588
Macrobiotus shonaicus Japan			LC431598	LC431589
Macrobiotus shonaicus Japan			LC431599	LC431590

Macrobiotus hannae Poland	MH063922	MH063924		
Macrobiotus papei Tanzania	MH063881	MH063880	MH063921	MH057763
Macrobiotus canaricus Spain	MH063925	MH063934		
Macrobiotus noongaris Australia	MK737069	MK737063	MK737065	MK737919
Macrobiotus noongaris Australia			MK737066	
Macrobiotus kamilae India	MK737070	MK737064	MK737067	MK737920
Macrobiotus kamilae India				MK737920
Macrobiotus crustulus French Guiana	MT261912	MT261903		
Macrobiotus basiatus USA	MT498094	MT488397		
Macrobiotus sottilei Poland	MW247178	MW247175	MW247179	MW246133
Macrobiotus glebkai Ukraine	MW247177	MW247176		
Macrobiotus margoae USA	MN888368	MN888354		
Macrobiotus margoae USA	MT809072	MT809084		
Macrobiotus margoae USA	MT809073	MT809085		
Macrobiotus ripperi Poland	MN888367	MN888353		
Macrobiotus ripperi Poland	MT809074	MT809086		
Macrobiotus ripperi Finland	MN888366	MN888352		
Macrobiotus ripperi Finland	MT809075	MT809088		
Macrobiotus ripperi Finland	MT809076	MT809089		
Macrobiotus rybaki Greece	MW588029	MW588034		
Macrobiotus rybaki Greece	MW588028	MW588035		
Macrobiotus wandae Nepal	MN435109	MN435114		
Macrobiotus wandae Nepal	MN435110	MN435118		
Macrobiotus wandae Nepal	MN435111	MN435119		
Macrobiotus wandae Nepal	MN435112	MN435116		
Macrobiotus wandae Nepal	MN435113	MN435117		
Macrobiotus sandrae Germany	MW695445			
Macrobiotus sandrae Germany	MW695446			
Macrobiotus azzunae Tunisia	MW695447	MW695450		
Macrobiotus azzunae Tunisia	MW695448	MW695451		
Macrobiotus azzunae Tunisia	MW695449	MW695452		
Macrobiotus birendrai Canada	MW680641			
Macrobiotus ariekammensis groenlandicus Greenland	MZ463662			
Macrobiotus ariekammensis groenlandicus Greenland	MZ463663			
Macrobiotus ariekammensis groenlandicus Greenland	MZ463664			
Macrobiotus ariekammensis ariekammensis Norway	MZ463668			
Macrobiotus ariekammensis ariekammensis Norway	MZ463669			
Macrobiotus ariekammensis ariekammensis Norway	MZ463670			
Macrobiotus kirghizicus Kyrgyzstan	MZ463665			
Macrobiotus kirghizicus Kyrgyzstan	MZ463666			
Macrobiotus kirghizicus Kyrgyzstan	MZ463667			
Macrobiotus sp.	EF632478			
Macrobiotus sp.	EF632479			
Macrobiotus sp.	EF632480			
Macrobiotus gr. *hufelandi*	EF632481			
Macrobiotus gr. *hufelandi*	EF632482			
Macrobiotus gr. *hufelandi*	EF632483			
Macrobiotus gr. *hufelandi*	EF632484			

Macrobiotus gr. *hufelandi*	EF632485	
Macrobiotus gr. *hufelandi*	EF632486	
Macrobiotus gr. *hufelandi*	EF632487	
Macrobiotus gr. *hufelandi*	EF632488	
Macrobiotus gr. *hufelandi*	EF632489	
Macrobiotus gr. *hufelandi*	EF632490	
Macrobiotus gr. *hufelandi*	EF632491	
Macrobiotus sp.	EU266935	
Macrobiotus sp.	EU266936	
Macrobiotus sp.	EU266937	
Macrobiotus sp.	EU266938	
Macrobiotus sp.	U49912	
Macrobiotus sp. Austria	MN927135	
Xerobiotus pseudohufelandi Italy	HQ604989	
Xerobiotus pseudohufelandi Italy	HQ604990	
Xerobiotus gretae Sweden	MW588434	MW588438
Xerobiotus gretae Sweden	MW588435	MW588439
Xerobiotus gretae Sweden	MW588436	MW588440
Xerobiotus gretae Sweden	MW588437	MW588441
Xerobiotus sp. Poland	MN888373	MN888358
Xerobiotus sp. South Africa	MN888374	MN888359
Mesobiotus hilariae Antarctica	KT226068	
Mesobiotus hilariae Antarctica	KT226069	
Mesobiotus hilariae Antarctica	KT226070	KT226079
Mesobiotus hilariae Antarctica	KT226071	
Mesobiotus cf. *mottai* Antarctica	KT226072	KT226079
Mesobiotus polaris Antarctica	KT226075	
Mesobiotus polaris Antarctica	KT226076	
Mesobiotus polaris Antarctica	KT226077	KT226087
Mesobiotus polaris Antarctica	KT226078	
Mesobiotus gr. *harmsworthi* Italy	HQ604967	HQ604981
Mesobiotus gr. *harmsworthi* Italy	HQ604968	HQ604982
Mesobiotus gr. *harmsworthi* Italy	HQ604969	HQ604985
Mesobiotus gr. *harmsworthi* Italy	HQ604970	HQ604986
Mesobiotus gr. *harmsworthi* Italy	KT226073	KT226083
Mesobiotus gr. *harmsworthi* Italy	KT226074	KT226084
Mesobiotus furciger Antarctica	EU266927	
Mesobiotus furciger Antarctica	EU266928	
Mesobiotus furciger Antarctica	EU266929	
Mesobiotus sp. Antarctica	EU266926	
Mesobiotus cf. *furciger*	EF632476	
Mesobiotus cf. *furciger*	EF632475	
Mesobiotus cf. *furciger*	EF632474	
Mesobiotus cf. *furciger*	EF632473	
Mesobiotus cf. *furciger*	EF632472	
Mesobiotus cf. *furciger*	EF632471	
Mesobiotus cf. *furciger*	EF632470	
Mesobiotus cf. *furciger*	EF632469	
Mesobiotus sp. Antarctica	JX296290	
Mesobiotus cf. *furciger* Antarctica	JX296285	
Mesobiotus cf. *furciger* Antarctica	JX296284	
Mesobiotus cf. *furciger* Antarctica	JX296283	

Mesobiotus cf. *furciger* Antarctica	JX296282	
Mesobiotus cf. *furciger* Antarctica	JX296281	
Mesobiotus cf. *furciger* Antarctica	JX296280	
Mesobiotus cf. *furciger* Antarctica	JX296279	
Mesobiotus cf. *furciger* Antarctica	JX296278	
Mesobiotus cf. *furciger* Antarctica	JX296277	
Mesobiotus cf. *furciger* Antarctica	JX296332	
Mesobiotus sp.	JX296334	
Mesobiotus cf. *furciger* Antarctica	JX296329	
Mesobiotus cf. *furciger* Antarctica	JX296328	
Mesobiotus cf. *furciger* Antarctica	JX296327	
Mesobiotus cf. *furciger* Antarctica	JX296326	
Mesobiotus cf. *furciger* Antarctica	JX296324	
Mesobiotus cf. *furciger* Antarctica	JX296323	
Mesobiotus cf. *furciger* Antarctica	JX296321	
Mesobiotus cf. *furciger* Antarctica	JX296317	
Mesobiotus cf. *furciger* Antarctica	JX296316	
Mesobiotus cf. *furciger* Antarctica	JX296315	
Mesobiotus cf. *furciger* Antarctica	JX296314	
Mesobiotus cf. *furciger* Antarctica	JX296312	
Mesobiotus cf. *furciger* Antarctica	JX296311	
Mesobiotus cf. *furciger* Antarctica	JX296309	
Mesobiotus cf. *furciger* Antarctica	JX296308	
Mesobiotus cf. *furciger* Antarctica	JX296307	
Mesobiotus cf. *furciger* Antarctica	JX296306	
Mesobiotus cf. *furciger* Antarctica	JX296305	
Mesobiotus cf. *furciger* Antarctica	JX296303	
Mesobiotus cf. *furciger* Antarctica	JX296302	
Mesobiotus cf. *furciger* Antarctica	JX296300	
Mesobiotus cf. *furciger* Antarctica	JX296299	
Mesobiotus cf. *furciger* Antarctica	JX296298	
Mesobiotus cf. *furciger* Antarctica	JX296297	
Mesobiotus cf. *furciger* Antarctica	JX296296	
Mesobiotus cf. *furciger* Antarctica	JX296295	
Mesobiotus cf. *furciger* Antarctica	JX296292	
Mesobiotus cf. *furciger* Antarctica	JX296291	
Mesobiotus cf. *furciger* Antarctica	JX296289	
Mesobiotus cf. *furciger* Antarctica	JX296288	
Mesobiotus cf. *furciger* Antarctica	JX296287	
Mesobiotus cf. *furciger* Antarctica	JX296338	
Mesobiotus cf. *furciger* Antarctica	JX296337	
Mesobiotus cf. *furciger* Antarctica	JX296336	
Mesobiotus cf. *furciger* Antarctica	JX296335	
Mesobiotus philippinicus Philippines	KX129793	KX129794
Mesobiotus insanis Philippines	MF441488	MF441489
Mesobiotus ethiopicus Ethiopia	MF678793	MF678792
Mesobiotus romani Ecuador	MH197158	MH197151
Mesobiotus fiedleri Madagascar	MH681585	MH681593
Mesobiotus datanlanicus Vietnam	MK584659	MK584658
Mesobiotus dilimanensis Philippines	MN257048	MN257049
Mesobiotus cf. *barabanovi* Kyrgyzstan	MN310392	MN310388
Mesobiotus anastasiae South Africa	MT903468	MT903612
Mesobiotus imperialis Vietnam	OL257854	
Mesobiotus imperialis Vietnam	OL257855	

Mesobiotus marmoreus Vietnam	OL257856	
Mesobiotus marmoreus Vietnam	OL257857	
Mesobiotus marmoreus Vietnam	OL257858	
Mesobiotus furciger Antarctica	MW751933	
Mesobiotus furciger Antarctica	MW751934	
Mesobiotus furciger Antarctica	MW751935	
Mesobiotus furciger Antarctica	MW751936	
Mesobiotus furciger Antarctica	MW751937	
Mesobiotus furciger Antarctica	MW751938	
Mesobiotus furciger Antarctica	MW751939	
Mesobiotus sp. stellate egg Antarctica	MW751940	
Mesobiotus sp. stellate egg Antarctica	MW751941	
Mesobiotus sp. stellate egg Antarctica	MW751942	
Mesobiotus furciger Antarctica	MW751943	
Mesobiotus furciger Antarctica	MW751944	
Mesobiotus furciger Antarctica	MW751945	
Mesobiotus furciger Antarctica	MW751946	
Mesobiotus furciger Antarctica	MW751947	
Mesobiotus furciger Antarctica	MW751948	
Mesobiotus furciger Antarctica	MW751949	
Mesobiotus furciger Antarctica	MW751950	
Mesobiotus furciger Antarctica	MW751951	
Mesobiotus furciger Antarctica	MW751952	
Mesobiotus furciger Antarctica	MW751953	
Mesobiotus furciger Antarctica	MW751954	
Mesobiotus furciger Antarctica	MW751955	
Mesobiotus furciger Antarctica	MW751956	
Mesobiotus furciger Antarctica	MW751957	
Mesobiotus furciger Antarctica	MW751958	
Mesobiotus furciger Antarctica	MW751959	
Mesobiotus furciger Antarctica	MW751960	
Mesobiotus furciger Antarctica	MW751961	
Mesobiotus furciger Antarctica	MW751962	
Mesobiotus furciger Antarctica	MW751963	
Mesobiotus furciger Antarctica	MW751964	
Mesobiotus furciger Antarctica	MW751965	
Mesobiotus furciger Antarctica	MW751966	
Mesobiotus furciger Antarctica	MW751967	
Mesobiotus furciger Antarctica	MW751968	
Mesobiotus furciger Antarctica	MW751969	
Minibiotus furcatus Spain	FJ435745	FJ435759
Minibiotus furcatus Spain	FJ435746	FJ435760
Minibiotus furcatus Spain	FJ435747	FJ435758
Minibiotus furcatus Italy	HQ604977	
Minibiotus furcatus Italy	HQ604978	
Minibiotus gumersindoi Spain	FJ435748	FJ435761
Minibiotus gr. *intermedius* Italy	HQ604979	
Minibiotus gr. *intermedius* Italy	HQ604980	
Minibiotus ioculator South Africa	MT023998	MT024041
Minibiotus pentannulatus Tanzania	MT023999	MT024042
Minibiotus sp.	EU266932	
Minibiotus sp.	EU266933	
Minibiotus sp.	EU266934	

Paramacrobiotus areolatus Norway	MH664931	MH664948		
Paramacrobiotus areolatus Croatia	DQ839602			
Paramacrobiotus tonollii USA	DQ839605			
Paramacrobiotus tonollii USA	MH664946	MH664963		
Paramacrobiotus lachowskae Colombia	MF568532	MF568533		
Paramacrobiotus gr. *richtersi* Germany	DQ839603			
Paramacrobiotus kenianus Kenya	EU038081			
Paramacrobiotus kenianus Kenya	EU038080			
Paramacrobiotus fairbanksi USA	EU038078		GQ403674	
Paramacrobiotus gr. *richtersi* Spain	FJ435744			
Paramacrobiotus gr. *richtersi* Spain	FJ435743	FJ435757		
Paramacrobiotus richtersi Ireland	HQ604986			MK040992
Paramacrobiotus richtersi Ireland	MK041023			
Paramacrobiotus spatialis Italy	HQ604985			
Paramacrobiotus spatialis Italy	MK041024			
Paramacrobiotus spatialis Italy	MK041025			
Paramacrobiotus aff. *spatialis* Ca1 Guidetti et al. 2019 Italy	MK041026			
Paramacrobiotus fairbanksi Italy	MK041027			
Paramacrobiotus fairbanksi Italy	MK041028			
Paramacrobiotus fairbanksi Italy	MK041029			
Paramacrobiotus fairbanksi Poland	MH664941	MH664950		
Paramacrobiotus fairbanksi Poland	MH664942	MH664959		
Paramacrobiotus fairbanksi Antarctica	MN960302	MN960306		
Paramacrobiotus fairbanksi Antarctica	MN960303	MN960307		
Paramacrobiotus fairbanksi Antarctica	MN960304			
Paramacrobiotus depressus Italy	MK041030			
Paramacrobiotus celsus Italy	MK041031			
Paramacrobiotus arduus Italy	MK041032			
Paramacrobiotus filipi Malaysia	MT261913	MT261904		
Paramacrobiotus experimentalis Madagascar	MN073467	MN073466		
Paramacrobiotus experimentalis Madagascar	MN073468	MN073465		
Paramacrobiotus sp. Australia	MH664932	MH664949		
Paramacrobiotus sp. Tanzania	MH664933	MH664951		
Paramacrobiotus sp. Brazil	MH664934	MH664952		
Paramacrobiotus sp. France	MH664935	MH664953		
Paramacrobiotus sp. Hungary	MH664936	MH664954		
Paramacrobiotus sp. Italy	MH664937	MH664955		
Paramacrobiotus sp. Madagascar	MH664938	MH664956		
Paramacrobiotus sp. Norway	MH664939	MH664957		
Paramacrobiotus sp. New Zealand	MH664940	MH664958		
Paramacrobiotus sp. Portugal	MH664943	MH664960		
Paramacrobiotus sp. Portugal	MH664944	MH664961		
Paramacrobiotus sp. Tunisia	MH664945	MH664962		
Paramacrobiotus sp.	EF632477			
Paramacrobiotus sp.	X81442			
Sisubiotus spectabilis Finland	MN888371	MN888357		
Sisubiotus spectabilis Norway	MN888372	MN888364		
Tenuibiotus voronkovi Arctic	KX810045	KX810049		

Tenuibiotus zandrae Greenland	MN443040	MN443035
Tenuibiotus cf. *ciprianoi* Spain	MN888376	MN888361
Tenuibiotus danilovi Kyrgyzstan	MN888377	MN888362
Tenuibiotus tenuiformis Kyrgyzstan	MN888378	MN888363
Adorybiotus granulatus Norway	HQ604961	
Adorybiotus granulatus Norway	HQ604962	
Adorybiotus cf. *granulatus* Japan	MT812475	MT812464
Crenubiotus sp. Scotland	MT812473	
Crenubiotus crenulatus Norway	MT812474	
Crenubiotus sp. Greenland	OM179850	
Crenubiotus sp. Greenland	OM179851	
Crenubiotus ruhesteini Germany	MW074384	MW074390
Crenubiotus ruhesteini Germany	MW074385	MW074391
Crenubiotus ruhesteini Germany	MW074386	MW074392
Crenubiotus ruhesteini Germany	MW074387	MW074393
Crenubiotus ruhesteini Germany	MW074388	MW074394
Crenubiotus ruhesteini Germany	MW074389	MW074395
Diaforobiotus islandicus Norway	HQ604972	KT778704
Diaforobiotus islandicus Norway	HQ604973	KT778705
Diaforobiotus islandicus Iceland	MT812470	MT812462
Diaforobiotus sp. Norway	MT812471	MT812463
Diaforobiotus sp. Indonesia	MT812472	MT812464
Diaforobiotus hyperonyx Italy	OM179852	
Diaforobiotus hyperonyx Italy	OM179853	
Diaforobiotus hyperonyx Italy	OM179854	
Diaforobiotus hyperonyx Italy	OM179855	
Richtersius coronifer Norway	MH681760	MH681757
Richtersius cf. *coronifer* Greenland	KT778712	KT778702
Richtersius cf. *coronifer* Greenland	KT778713	KT778703
Richtersius cf. *coronifer* Italy	HQ604987	HQ604695
Richtersius cf. *coronifer* Italy	HQ604988	HQ604696
Richtersius cf. *coronifer* Italy	KT778706	KT778697
Richtersius cf. *coronifer* Italy	KT778707	KT778698
Richtersius cf. *coronifer* Mongolia	KT778708	KT778699
Richtersius cf. *coronifer* Mongolia	KT778709	KT778700
Richtersius cf. *coronifer* Mongolia	KT778710	KT778701
Richtersius cf. *coronifer* Italy	KT778711	
Richtersius aff. *coronifer* Italy	MH681761	MH681758
Richtersius aff. *coronifer* Poland	MH681762	MH681759
Richtersius tertius Greece	MK211386	MK211384
Richtersius aff. *coronifer* Italy	MK211387	MK211385
Richtersius ziemowiti Nepal	MT241891	MT241895
Murrayon pullari Italy	HQ604983	
Murrayon pullari Italy	HQ604984	
Murrayon cf. *pullari* Italy	MT812477	MT812465
Murrayon pullari Greenland	GQ849026	MH079499
Murrayon dianeae Italy	HQ604981	
Murrayon dianeae Italy	HQ604982	
Murrayon cf. *dianeae* Spain	FJ435737	FJ435762

Dactylobiotus ovimutans Antarctica	MT136805	MT136807
Dactylobiotus ambiguus	GQ925676	MH079500
Dactylobiotus ambiguus	GQ925677	
Dactylobiotus ambiguus	GQ925680	
Dactylobiotus ambiguus	GQ925681	
Dactylobiotus octavi Greenland	GQ849025	GQ849049
Dactylobiotus selenicus Finland	MT812476	MT812466
Dactylobiotus parthenogeneticus Poland	MT373695	MT373701
Dactylobiotus parthenogeneticus France	MT373694	MT373700
Dactylobiotus parthenogeneticus UK	MT373693	MT373699
