# Supplementary material for: Resistance to Extreme Stresses by a Newly Discovered Japanese Tardigrade Species, Macrobiotus kyoukenus (Eutardigrada, Macrobiotidae)
Source: Insects. 2022 Jul 15;13(7):634. doi: 10.3390/insects13070634 (PMC9315711; doi:10.3390/insects13070634)
Supplement: Supplementary file 1 [file insects-13-00634-s001.zip › Cesari et al Table S3.pdf]

Table S3. *cox1* gene genetic distances (p-distance) computed among *Macrobiotus* specimens pertaining to the subclade C (*sensu* [29]). The analysis was carried out on a 657 bp dataset.

		1	2	3	4	5	6	7	8	9	10	11	12	13	14	15	16	17	18	19	20	21	22	23	24	25	26	27	28	29	30	31
1	MK040992 *Paramacrobiotus richtersi*																															
2	MG757136 *Macrobiotus shonaicus* Tsuruoka Japan	0.246																														
3	MG757137 *Macrobiotus shonaicus* Tsuruoka Japan	0.244	0.011																													
4	LC431582 *Macrobiotus* cf. *shonaicus* Oritsume Japan	0.247	0.013	0.005																												
5	LC431583 *Macrobiotus* cf. *shonaicus* Hiyoshi Japan	0.248	0.003	0.011	0.013																											
6	LC431584 *Macrobiotus* cf. *shonaicus* Kamiyoshida Japan	0.245	0.008	0.003	0.008	0.011																										
7	LC431585 *Macrobiotus* cf. *shonaicus* Shizuoka Japan	0.245	0.008	0.003	0.008	0.011	0.007																									
8	LC431586 *Macrobiotus* cf. *shonaicus* Kyoto Japan	0.247	0.000	0.008	0.013	0.003	0.008	0.008																								
9	LC431587 *Macrobiotus* cf. *shonaicus* Rokko Japan	0.247	0.010	0.002	0.007	0.013	0.005	0.005	0.010																							
10	LC431588 *Macrobiotus* cf. *shonaicus* Bizan Japan	0.250	0.003	0.011	0.016	0.007	0.011	0.011	0.003	0.013																						
11	LC431589 *Macrobiotus* cf. *shonaicus* Oki Japan	0.245	0.008	0.000	0.005	0.011	0.003	0.003	0.008	0.002	0.011																					
12	LC431590 *Macrobiotus* cf. *shonaicus* Kagoshima Japan	0.247	0.010	0.002	0.007	0.013	0.005	0.005	0.010	0.000	0.013	0.002																				
13	MH057763 *Macrobiotus papei* Tanzania	0.252	0.217	0.217	0.214	0.217	0.220	0.219	0.219	0.217	0.222	0.219	0.217																			
14	KY797267 *Macrobiotus scoticus* Scotland	0.248	0.219	0.221	0.222	0.219	0.219	0.217	0.219	0.219	0.222	0.220	0.219	0.184																		
15	KT951668 *Macrobiotus paulinae* Kenya	0.233	0.220	0.222	0.219	0.222	0.217	0.219	0.221	0.222	0.224	0.221	0.222	0.202	0.205																	
16	KX810011 *Macrobiotus polypiformis* Ecuador	0.251	0.196	0.199	0.197	0.196	0.196	0.196	0.192	0.197	0.196	0.196	0.197	0.235	0.229	0.203																
17	KX810012 *Macrobiotus polypiformis* Ecuador	0.254	0.199	0.202	0.201	0.199	0.199	0.199	0.196	0.201	0.199	0.199	0.201	0.240	0.226	0.207	0.029															
18	KC193572 *Macrobiotus kristenseni* Argentina	0.268	0.213	0.211	0.209	0.209	0.209	0.209	0.211	0.213	0.215	0.211	0.213	0.224	0.208	0.213	0.229	0.233														
19	KC193573 *Macrobiotus kristenseni* Argentina	0.269	0.214	0.214	0.212	0.212	0.212	0.212	0.214	0.216	0.218	0.214	0.216	0.228	0.212	0.212	0.230	0.234	0.000													
20	KC193574 *Macrobiotus kristenseni* Argentina	0.259	0.220	0.222	0.219	0.219	0.219	0.219	0.219	0.219	0.223	0.221	0.219	0.209	0.206	0.208	0.230	0.235	0.014	0.014												
21	KC193575 *Macrobiotus kristenseni* Argentina	0.259	0.220	0.220	0.217	0.217	0.217	0.217	0.217	0.217	0.220	0.219	0.217	0.209	0.204	0.208	0.228	0.232	0.014	0.014	0.000											
22	KC193576 *Macrobiotus kristenseni* Argentina	0.259	0.220	0.220	0.217	0.217	0.217	0.217	0.217	0.217	0.220	0.219	0.217	0.209	0.204	0.208	0.228	0.232	0.014	0.014	0.000	0.000										
23	MK737919 *Macrobiotus noongaris* Australia	0.241	0.204	0.207	0.202	0.199	0.197	0.199	0.197	0.202	0.201	0.201	0.202	0.237	0.215	0.214	0.213	0.218	0.215	0.214	0.232	0.232	0.232									
24	MK737920 *Macrobiotus kamilae* India	0.246	0.222	0.224	0.227	0.222	0.223	0.230	0.223	0.225	0.227	0.227	0.225	0.221	0.226	0.212	0.207	0.209	0.241	0.242	0.245	0.242	0.242	0.225								
25	MK737921 *Macrobiotus kamilae* India	0.246	0.222	0.224	0.227	0.222	0.223	0.230	0.223	0.225	0.227	0.227	0.225	0.221	0.226	0.212	0.207	0.209	0.241	0.242	0.245	0.242	0.242	0.227	0.002							
26	MW246133 *Macrobiotus sottilei* Poland	0.264	0.192	0.193	0.196	0.196	0.196	0.192	0.192	0.197	0.196	0.196	0.197	0.211	0.210	0.210	0.210	0.210	0.221	0.224	0.229	0.226	0.226	0.209	0.215	0.215						
27	**C4313 V2 COI**	**0.271**	**0.209**	**0.209**	**0.206**	**0.209**	**0.209**	**0.204**	**0.206**	**0.206**	**0.209**	**0.207**	**0.206**	**0.204**	**0.221**	**0.212**	**0.204**	**0.212**	**0.249**	**0.253**	**0.244**	**0.241**	**0.241**	**0.223**	**0.218**	**0.218**	**0.204**					
28	**C4313 V3 COI**	**0.276**	**0.209**	**0.212**	**0.207**	**0.210**	**0.210**	**0.206**	**0.207**	**0.207**	**0.210**	**0.209**	**0.207**	**0.205**	**0.221**	**0.214**	**0.209**	**0.216**	**0.245**	**0.246**	**0.240**	**0.239**	**0.239**	**0.228**	**0.218**	**0.218**	**0.209**	**0.006**				
29	**C4313 US3 COI**	**0.276**	**0.209**	**0.212**	**0.207**	**0.210**	**0.210**	**0.206**	**0.207**	**0.207**	**0.210**	**0.209**	**0.207**	**0.206**	**0.222**	**0.215**	**0.210**	**0.218**	**0.245**	**0.246**	**0.240**	**0.239**	**0.239**	**0.229**	**0.219**	**0.219**	**0.209**	**0.006**	**0.000**			
30	**C4313 US4 COI**	**0.276**	**0.207**	**0.211**	**0.207**	**0.210**	**0.210**	**0.206**	**0.207**	**0.207**	**0.210**	**0.209**	**0.207**	**0.206**	**0.222**	**0.215**	**0.209**	**0.217**	**0.245**	**0.246**	**0.240**	**0.239**	**0.239**	**0.228**	**0.218**	**0.218**	**0.209**	**0.006**	**0.000**	**0.000**		
31	**C4313 US5 COI**	**0.273**	**0.209**	**0.209**	**0.204**	**0.208**	**0.208**	**0.203**	**0.204**	**0.204**	**0.208**	**0.206**	**0.204**	**0.203**	**0.220**	**0.211**	**0.204**	**0.212**	**0.249**	**0.253**	**0.243**	**0.240**	**0.240**	**0.222**	**0.216**	**0.216**	**0.206**	**0.000**	**0.006**	**0.006**	**0.006**	
32	**C4313 US8 COI**	**0.272**	**0.205**	**0.206**	**0.206**	**0.209**	**0.209**	**0.204**	**0.206**	**0.206**	**0.209**	**0.207**	**0.206**	**0.205**	**0.219**	**0.210**	**0.200**	**0.208**	**0.253**	**0.255**	**0.247**	**0.245**	**0.245**	**0.219**	**0.218**	**0.218**	**0.206**	**0.002**	**0.006**	**0.006**	**0.006**	**0.002**
