# Supplementary material for: Resistance to Extreme Stresses by a Newly Discovered Japanese Tardigrade Species, Macrobiotus kyoukenus (Eutardigrada, Macrobiotidae)
Source: Insects. 2022 Jul 15;13(7):634. doi: 10.3390/insects13070634 (PMC9315711; doi:10.3390/insects13070634)
Supplement: Supplementary file 1 [file insects-13-00634-s001.zip › Cesari et al Table S4.pdf]

Table S4. ITS-2gene genetic distances (p-distance) computed among *Macrobiotus* specimens pertaining to the subclade C (*sensu* [29]). The analysis was carried out on a 530 bp dataset.

		1	2	3	4	5	6	7	8	9	10	11	12	13	14	15	16	17	18	19	20	21	22	23	24	25	26
1	GQ403674 *Paramacrobiotus fairbanksi* Alaska USA																										
2	GQ403680 *Macrobiotus sapiens* Croatia	0.461																									
3	MG757134 *Macrobiotus shonaicus* Tsuruoka Japan	0.437	0.160																								
4	MG757135 *Macrobiotus shonaicu*s Tsuruoka Japan	0.441	0.160	0.006																							
5	LC431591 *Macrobiotus* cf. *shonaicus* Oritsume Japan	0.467	0.170	0.006	0.012																						
6	LC431592 *Macrobiotus* cf. *shonaicus* Hiyoshi Japan	0.460	0.177	0.009	0.015	0.011																					
7	LC431593 *Macrobiotus* cf. *shonaicus* Kamiyoshida Japan	0.474	0.173	0.009	0.003	0.011	0.013																				
8	LC431594 *Macrobiotus* cf. *shonaicus* Shizuoka Japan	0.467	0.176	0.012	0.006	0.013	0.015	0.007																			
9	LC431595 *Macrobiotus* cf. *shonaicus* Kyoto Japan	0.466	0.167	0.012	0.018	0.013	0.011	0.015	0.017																		
10	LC431596 *Macrobiotus* cf. *shonaicus* Rokko Japan	0.472	0.170	0.009	0.015	0.013	0.011	0.015	0.017	0.004																	
11	LC431597 *Macrobiotus* cf. *shonaicus* Bizan Japan	0.474	0.170	0.012	0.006	0.015	0.017	0.004	0.011	0.020	0.020																
12	LC431598 *Macrobiotus* cf. *shonaicus* Oki Japan	0.470	0.177	0.012	0.006	0.020	0.022	0.013	0.015	0.024	0.024	0.017															
13	LC431599 *Macrobiotus* cf. *shonaicus* Kagoshima Japan	0.463	0.167	0.006	0.012	0.009	0.007	0.011	0.013	0.009	0.009	0.015	0.020														
14	MH063921 *Macrobiotus papei* Tanzania	0.455	0.086	0.123	0.120	0.122	0.122	0.117	0.119	0.120	0.117	0.114	0.120	0.115													
15	KY797268 *Macrobiotus scoticus* Scotland	0.543	0.255	0.259	0.259	0.213	0.213	0.216	0.218	0.207	0.210	0.216	0.216	0.216	0.215												
16	KT935500 *Macrobiotus paulinae* Kenya	0.497	0.186	0.164	0.158	0.135	0.135	0.128	0.131	0.128	0.128	0.128	0.131	0.128	0.132	0.228											
17	KX810010 *Macrobiotus polypiformis* Ecuador	0.477	0.301	0.208	0.204	0.213	0.213	0.210	0.213	0.203	0.206	0.208	0.214	0.208	0.220	0.266	0.211										
18	MK737065 *Macrobiotus noongaris* Australia	0.465	0.191	0.149	0.149	0.171	0.162	0.169	0.171	0.159	0.156	0.166	0.172	0.159	0.165	0.249	0.174	0.252									
19	MK737066 *Macrobiotus noongaris* Australia	0.462	0.191	0.143	0.143	0.166	0.156	0.164	0.166	0.154	0.151	0.161	0.167	0.154	0.165	0.252	0.171	0.252	0.010								
20	MK737067 *Macrobiotus kamilae* India	0.487	0.176	0.151	0.151	0.143	0.143	0.140	0.143	0.134	0.132	0.137	0.140	0.134	0.131	0.270	0.124	0.244	0.152	0.146							
21	MW247179 *Macrobiotus sottilei* Poland	0.464	0.078	0.102	0.102	0.108	0.113	0.110	0.113	0.111	0.108	0.108	0.116	0.105	0.068	0.235	0.129	0.244	0.139	0.133	0.126						
22	**C4313 V3 ITS2**	**0.456**	**0.133**	**0.143**	**0.143**	**0.137**	**0.137**	**0.135**	**0.137**	**0.135**	**0.131**	**0.135**	**0.140**	**0.131**	**0.096**	**0.217**	**0.109**	**0.227**	**0.181**	**0.176**	**0.122**	**0.104**					
23	**C4313 US3 ITS2**	**0.456**	**0.136**	**0.140**	**0.140**	**0.135**	**0.135**	**0.133**	**0.135**	**0.133**	**0.129**	**0.133**	**0.138**	**0.129**	**0.098**	**0.217**	**0.111**	**0.225**	**0.181**	**0.176**	**0.122**	**0.107**	**0.002**				
24	**C4313 US4 ITS2**	**0.456**	**0.133**	**0.143**	**0.143**	**0.137**	**0.137**	**0.135**	**0.137**	**0.135**	**0.131**	**0.135**	**0.140**	**0.131**	**0.096**	**0.217**	**0.109**	**0.227**	**0.181**	**0.176**	**0.122**	**0.104**	**0.000**	**0.002**			
25	**C4313 US5 ITS2**	**0.456**	**0.136**	**0.140**	**0.140**	**0.135**	**0.135**	**0.133**	**0.135**	**0.133**	**0.129**	**0.133**	**0.138**	**0.129**	**0.098**	**0.217**	**0.111**	**0.225**	**0.181**	**0.176**	**0.122**	**0.107**	**0.002**	**0.000**	**0.002**		
26	**C4313 US6 ITS2**	**0.456**	**0.133**	**0.143**	**0.143**	**0.137**	**0.137**	**0.135**	**0.137**	**0.135**	**0.131**	**0.135**	**0.140**	**0.131**	**0.096**	**0.217**	**0.109**	**0.227**	**0.181**	**0.176**	**0.122**	**0.104**	**0.000**	**0.002**	**0.000**	**0.002**	
27	**C4313 US7 ITS2**	**0.456**	**0.133**	**0.143**	**0.143**	**0.137**	**0.137**	**0.135**	**0.137**	**0.135**	**0.131**	**0.135**	**0.140**	**0.131**	**0.096**	**0.217**	**0.109**	**0.227**	**0.181**	**0.176**	**0.122**	**0.104**	**0.000**	**0.002**	**0.000**	**0.002**	**0.000**
